# Development and Validation of the Sexual Minority Adolescent Rejection Sensitivity Scale

**DOI:** 10.1007/s10508-022-02474-6

**Published:** 2022-12-01

**Authors:** Wouter J. Kiekens, Laura Baams, Brian A. Feinstein, René Veenstra

**Affiliations:** 1 Department of Sociology/Interuniversity Center for Social Science Theory and Methodology (ICS), University of Groningen, Grote Kruisstraat 2/1, 9712 TS Groningen, The Netherlands; 2 Department of Pedagogy and Educational Sciences, University of Groningen, Groningen, The Netherlands; 3 Department of Psychology, Rosalind Franklin University of Medicine and Science, North Chicago, IL USA

**Keywords:** Rejection sensitivity, Sexual minority, Adolescents, Mental health, Sexual orientation

## Abstract

Because no measure for sexual orientation-related rejection sensitivity (RS) for adolescents exists, we aimed to develop and validate the Sexual Minority Adolescent Rejection Sensitivity Scale (SMA-RSS). In Study 1, interviews with 22 sexual minority youth were conducted (*M* age = 18.86, *SD* = 3.03). Based on these interviews, 29 scenarios were developed as potential items for the SMA-RSS. In Study 2, exploratory factor analyses were conducted on these 29 scenarios in a sample of 397 sexual minority adolescents (*M* age = 16.63, *SD* = 1.07). The 14 best performing items were selected and a two-factor structure best fit the data. In Study 3, a confirmatory factor analysis was conducted and the test–retest reliability, criterion validity, convergent validity, and incremental validity of the SMA-RSS were assessed in a sample of 499 sexual minority adolescents (*M* age = 16.61, *SD* = 1.34). A bifactor model best fit the data and evidence was provided for a strong enough general factor to justify unidimensionality. For criterion validity, the SMA-RSS evidenced small to moderate correlations with minority stressors and mental health indicators. For convergent validity, we found a moderate correlation with general RS. For incremental validity, the SMA-RSS was associated with mental health indicators over and above minority stressors and general RS. Participants were moderately stable in their scores on the SMA-RSS over a one-month period. Taken together, the SMA-RSS captured unique situations in which sexual minority adolescents anxiously expect rejection and can aid in better understanding health disparities among sexual minority adolescents.

**Supplementary Information:**

The online version contains supplementary material available at 10.1007/s10508-022-02474-6.

## Introduction

Sexual minority adolescents report higher rates of internalizing problems than heterosexual adolescents such as depressive symptoms and anxiety symptoms (Fish et al., 2020; Plöderl & Tremblay, 2015). Experiences with minority stress can explain the higher rates of internalizing problems among sexual minority adolescents (Meyer, 2003). Here, minority stress is understood as additional stressors that sexual minority people experience related to their minority sexual orientation. These stressors exist on a continuum from distal (i.e., external stressors such as experiencing discrimination) to proximal (i.e., psychological processes such as expectations of rejection). Given the impact of minority stress on sexual minority adolescents’ mental health (Jackson & Mohr, 2016; Kiekens et al., 2020; Newcomb & Mustanski, 2010), it is essential to understand the mechanisms underlying these associations. This might also improve the design of prevention and intervention programs aimed at sexual minority adolescents.

Research has pointed to sexual orientation-related rejection sensitivity as a potential mechanism through which distal minority stressors affect mental health (Feinstein, 2020). Here, rejection sensitivity (RS) is understood as a process whereby people anxiously expect, readily perceive, and intensely react to sexual orientation-based rejection already in ambiguous situations where rejection is possible (Downey & Feldman, 1996; Feinstein, 2020). However, research on sexual orientation-related RS among sexual minority adolescents is scarce (Baams et al., 2020). A reason for the lack of research among sexual minority adolescents is that existing RS measures are population-specific and were developed for sexual minority adult men (Pachankis et al., 2008) and women (Dyar et al., 2016) and thus may not be developmentally appropriate for adolescents. To date, no sexual orientation-related RS measure has been developed for sexual minority adolescents. Therefore, we aimed to develop and validate a sexual orientation-related RS measure for sexual minority adolescents.

### Sexual Orientation-Related Rejection Sensitivity

Initially, research on RS focused on how early experiences of rejection with close significant others (e.g., parents) might lead to anxious expectations of rejection in subsequent close relationships (Downey & Feldman, 1996). This has been extended to rejection by unfamiliar others due to being a member of a stigmatized social group, which is referred to as status-based RS (Mendoza-Denton et al., 2002). Status-based RS acknowledges that direct or vicarious experiences of status-based stigma (e.g., mistreatment, exclusion) can lead to anxious expectations that status-based rejection will occur in the future. Status-based RS has been studied among different stigmatized social groups such as people of color (Mendoza-Denton et al., 2002) and women (London et al., 2012), as well as sexual minority adults (Dyar et al., 2016; Pachankis et al., 2008). For sexual minority groups, this is often referred to as sexual orientation-based RS.

As noted, minority stress theory includes expectations of rejection as a form of minority stress (Meyer, 2003). Sexual minority people often come to anticipate rejection and, in response, maintain vigilance for rejection-related cues. Because of this, expectations of rejection is described as a chronic stressor (Meyer, 2003). Although related, there is an essential distinction between sexual orientation-related RS and expectations of rejection. Expectations of rejection only capture a cognitive component, referring to the perceived likelihood of experiencing rejection. Sexual orientation-related RS similarly captures this cognitive component but includes an affective process as well, referring to the concern or anxiety that can come with expecting rejection (Feinstein, 2020). This is a critical distinction given that expectations of rejection are only weakly correlated with their accompanying affect (Zimmer-Gembeck et al., 2016). Thus, the combination of expecting rejection *and* being concerned or anxious about rejection triggers the sexual orientation-related RS dynamic, which may lead to adverse health consequences (Ayduk & Gyurak, 2008; Feinstein, 2020).

Sexual orientation-related RS was first studied in gay men, and for this purpose the Gay-Related Rejection Sensitivity Scale (GR-RSS) was developed (Pachankis et al., 2008). Because different populations anxiously expect rejection in different contexts, the Sexual Minority Women Rejection Sensitivity Scale (SMW-RSS) was developed to specifically study experiences of sexual orientation-related RS unique to sexual minority women (Dyar et al., 2016). Both the GR-RSS and the SMW-RSS are commonly used to study sexual orientation-related RS among sexual minority adults. In one set of studies, researchers found a direct association between sexual orientation-related RS and internalizing problems (Cohen et al., 2016; Feinstein et al., 2017). In another set of studies, researchers found that the associations between distal minority stressors and internalizing problems were mediated by RS (Dyar et al., 2018; Feinstein et al., 2012). Such studies demonstrate how sexual orientation-related RS can explain internalizing problems among sexual minority adults and how it can extend the minority stress framework to better understand minority stress processes in relation to internalizing problems. However, these findings may not be generalizable to sexual minority adolescents.

To our knowledge, RS has not been assessed in sexual minority adolescent samples. As noted, a potential reason for this is that no sexual orientation-related RS measure for adolescents has been developed. However, scholars have proposed that sexual orientation-related RS may be particularly important during this critical developmental period (Baams et al., 2020). Considering that different populations anxiously expect rejection in different contexts, sexual minority adolescents might experience minority stressors in different social contexts than sexual minority adults, making the existing RS measures developmentally inappropriate. For instance, an item from an existing RS measure describes a situation involving the principal at one’s child’s elementary school, which would be hard to relate to for adolescents (Dyar et al., 2016). Further, especially for sexual minority adolescents, research has underlined the importance of specific social contexts in which they might experience minority stressors (Goldbach & Gibbs, 2017). Thus, measures of RS for adolescents must reflect everyday social contexts that sexual minority adolescents traverse and in which they might anxiously expect rejection.

### The Present Study

Our goal was to develop and validate a measure of RS for sexual minority adolescents, the Sexual Minority Adolescent Rejection Sensitivity Scale (SMA-RSS). Three studies were conducted for the development and validation of this measure. First, interviews were conducted with sexual minority youth to identify situations in which adolescents might be concerned about the occurrence of sexual orientation-based rejection (Study 1). The findings from these interviews were used to develop scenarios relevant to sexual minority adolescents. Second, we administered an initial version of the measure based on these scenarios to a second sample of sexual minority adolescents, and we chose core scenarios based on the inter-item reliability and the factor structure of the measure (Study 2). Last, the factor structure was reassessed and the criterion validity, convergent validity, incremental validity, and test–retest reliability were examined in a third sample of sexual minority adolescents (Study 3).

## Study 1

To identify situations in which sexual minority adolescents might be concerned about the occurrence of sexual orientation-based rejection, we conducted interviews with sexual minority youth. The first aim was to identify situations in which sexual minority adolescents anxiously expect, readily perceive, and intensely react to sexual orientation-based rejection. The second aim was to investigate whether sexual minority adolescents could imagine and relate to the items from existing sexual orientation-related RS scales.

### Method

#### Participants

In total, 22 participants (50.0% assigned female at birth) were interviewed. Two participants had a minority gender identity but only reflected on experiences related to their sexual identity. The mean age was 18.86 (*SD* = 3.03), with 63.6% in the age range of 16–18. When asked how participants would describe their sexual identity, six participants identified as gay, one as gay but preferred no label, one as lesbian, three as lesbian but preferred no label, five as bisexual, one as bisexual but preferred no label, two as pansexual, one as pansexual but preferred no label, one as queer, and one used no label but reported same-gender attractions. All but one participant had disclosed their sexual identity to family and friends. Three participants had at least one parent who was not Dutch. Eight participants were in high school, five in vocational education, seven attended (applied) university, and two were working professionals. Concerning their living situation, 17 lived with their parent(s), three in student housing, one lived with their partner, and one lived independently.

#### Procedure

Semi-structured online video chat interviews were conducted. Participants were recruited using paid advertisements on Facebook and Instagram in September 2020 and January 2021. Although the aim of the study was to develop a measure for sexual minority adolescents, advertisements were targeted at 16–25-years-olds, who lived in the Netherlands, spoke Dutch, and had sexual minority-related interests on social media (e.g., Gay Pride Parade). The broader age range was chosen to facilitate online data collection. The limit was set at 25 because 25-year-olds can be considered young adults (Park et al., 2006), the developmental stage following adolescence, and might thus still be able to easily remember and reflect on their adolescent experiences. Youth interested in the study were directed to a website with more information on the study’s procedure. It was explained that participants would be interviewed in Dutch online through video chat for approximately one hour and that they would receive a €5 gift card after the interview. They were also informed that a research assistant would take detailed notes during the interview. The Ethics Committee of the Pedagogy and Educational Sciences Department of the University of Groningen approved procedures for all three studies in the current research.

#### Measures

##### Rejection Sensitivity Situations

Participants were first asked to describe a situation where they experienced explicit rejection because of their sexual orientation. Then, participants were asked whether they had ever experienced situations in which they felt concerned or worried that someone would reject them or treat them differently because of their sexual orientation. It was stressed that explicit rejection should not have necessarily occurred in these situations. To make sure that participants understood what kind of situations were meant, three examples were given from the interviewer’s (first author) personal experience in which he had felt concerned or worried that someone would reject him because of his sexual orientation. The interviewer then probed whether participants had experienced these situations in different social contexts that adolescents traverse, such as family, school, and friends. Participants older than 18 were asked to reflect on experiences during their high school years. During the interviews, participants were also able to reflect on vicarious experiences.

##### Existing Sexual Orientation-Related RS Scales

Participants were asked to react to 20 selected items from the GR-RSS and the SMW-RSS (Dyar et al., 2016; Pachankis et al., 2008). These items were translated to Dutch by the first author (native Dutch speaker, fluent in English) and subsequently checked by the second author (native Dutch speaker, fluent in English). They were explicitly asked whether they could imagine that the situation described in the item would happen to them and, if so, why. In selecting the 20 items, we excluded items that were not relevant for most adolescents (e.g., items assuming the participant had a child). Other items were slightly adjusted to be more relevant to adolescents (e.g., a group of coworkers was changed to a group of classmates), and some items were modified to be suitable for sexual minority adolescents of different genders (e.g., male partner was changed to same-sex partner). Further, across both measures there were items that were very similar. For these, only the most relevant item was used. Table S1A in the online supplementary provides an overview of the included items and how they were adjusted.

### Results

The first author read through all the interview notes and highlighted situations in which participants felt concerned or worried that they would be rejected or treated differently because of their sexual orientation. These situations were then categorized based on the type of perceived rejection that participants described. Examples of perceived rejection types were expecting negative remarks about one’s sexual orientation, feeling stared at, and feeling excluded. Within these perceived rejection types, the common social contexts and the common perpetrators were identified. Common social contexts were, for example, school, family, and shops. Perpetrators included schoolmates/classmates, family members (e.g., parents and siblings), and friends. Based on the rejection types, new scenarios were developed. If a rejection type was only relevant to a subset of sexual minority adolescents (e.g., only boys, only those who identified as gay), it was not developed into a scenario as the study’s goal was to create a measure applicable to all sexual minority adolescents. This resulted in the development of 15 scenarios.

For responses to the 20 items from the GR-RSS and the SMW-RSS, it was assessed whether participants could imagine that the situation described in the item would happen to them and whether they would relate the situation to their sexual orientation. Based on this, six items were deemed inappropriate for adolescents (e.g., an item about STI checks was inappropriate as adolescent participants in Study 1 had no experiences with STI checks). Four items had to be changed substantially to be appropriate for adolescents (e.g., because adolescent participants did not go on trains late at night, the late at night part was omitted from one item). Nine items needed slight wording changes (e.g., male/female friend was changed to a friend). One item required no adjustments (see Table S1A in the online supplementary material for an overview). These adjustments further confirmed that many of the items on existing scales were not developmentally appropriate for sexual minority adolescents. Some of the rejection types identified in the interviews corresponded to the items of the GR-RSS and the SMW-RSS. For these cases, we used the adapted items from the existing scales. This resulted in 14 scenarios from existing measures being adapted for sexual minority adolescents.

### Discussion

Based on 22 interviews, 29 scenarios (15 newly developed, 14 adapted from existing scales) were developed as potential items for the SMA-RSS. The social contexts and perpetrators identified by adolescent participants were not well represented in the existing scales, underlining that these scales were not developmentally appropriate for adolescents. Further, adolescent participants could not relate to a number of items on existing scales Overall, this initial measure of the SMA-RSS appears to capture unique situations in which sexual minority adolescents anxiously expect, readily perceive, and intensely react to sexual orientation-based rejection.

## Study 2

The aim of Study 2 was to test the 29 potential SMA-RSS items that were developed in Study 1 in a sample of sexual minority adolescents. To identify the best performing items and examine the factor structure, exploratory factor analyses (EFA) were conducted (Fabrigar & Wegener, 2011).

### Method

#### Participants

A total of 685 participants opened the survey. Of those, 42 were deemed ineligible to participate in the study based on not correctly answering three multiple-choice questions to check whether they fully understood what they were consenting to (described below), 233 did not complete any item about RS, 4 were duplicate cases, and 9 identified as heterosexual. Data from these participants were omitted from the sample, resulting in an analytic sample of 397. Such a sample size is considered acceptable for an EFA with communalities under and around 0.50 (the mean in our study was 0.38), a small number of factors (under 4), and an overdetermination of factors (6 items per factor, also the minimum in our study) (MacCallum et al., 1999). Sample demographics are presented in Table 1. Of note, sexual identity was assessed by asking “How do you describe your sexual orientation,” with response options lesbian, gay, bisexual, queer, pansexual, heterosexual, I am not sure, and other (specify). Gender identity was assessed by asking “How would you describe your gender identity,” with the following response options: man, woman, transman, transwoman, non-binary, genderqueer, gender fluid, I am not sure, and other (specify).”

**Table 1:** Demographic information by sex assigned at birth for Study 2 and Study 3

	Study 2 (*N* = 397)	Study 3 (*N* = 499)
	*n* (%)	*n* (%)
Age^a^	16.63 (1.07)	16.61 (1.36)
Sex assigned at birth		
Assigned male at birth	130 (32.7%)	172 (34.5%)
Assigned female at birth	267 (67.3%)	325 (65.1%)
Sexual identity^b^		
Gay	97 (24.4%)	118 (23.6%)
Lesbian	72 (18.1%)	84 (16.8%)
Bisexual	116 (29.2%)	141 (28.3%)
Queer	23 (5.8%)	36 (7.2%)
Pansexual	30 (7.6%)	45 (9.0%)
Asexual	13 (3.3%)	7 (1.4%)
Not sure	29 (7.3%)	18 (3.6%)
Another identity	16 (4.0%)	49 (9.8%)
Gender identity^b^		
Cisgender	292 (73.6%)	329 (66.0%)
Gender minority	105 (26.4%)	170 (34.0%)

^a^For age, *M* (*SD*) are given

^b^Numbers might not add up to the total sample size due to missing cases

#### Procedure

Participants were recruited using paid advertisements on Facebook and Instagram that ran in February and March 2021. The advertisements were targeted at 14–18 year olds, who lived in the Netherlands, spoke Dutch, and had sexual minority-related interests on social media (e.g., Gay Pride Parade). Adolescents interested in the study were directed to a website with more information on the study’s procedure. It was explained that participants would complete an online survey that would take approximately 10 min and that they could enter a raffle to win a €25 gift card. In both Study 2 and 3, a waiver for parental consent was obtained for participants younger than 16 years old. For all participants we used three multiple-choice questions to check whether they fully understood what they were consenting to (Nelson et al., 2019). Questions were: “What is expected from you when you participate in this research?,” “Where can you find support if you feel distressed after participating in this study?,” and “What are the potential risks of participating in this research?” If participants could not correctly answer all multiple-choice questions after three attempts, they were deemed ineligible to participate in the study. The questionnaire was administered in Dutch.

#### Measures

##### Potential SMA-RSS Items

Each of the 29 potential SMA-RSS items developed in Study 1 described a scenario in which sexual orientation-based rejection was possible. Participants received the items in random order. Participants started by reading the following introductory text: “We are now going to present you with several situations. They all start with ‘Imagine that…’. Try to imagine as best as possible what this situation would be like for you. After every situation, there are two questions. Some of the situations are about a same-sex partner; you can also read this as a same-gender partner.” After each item, participants were presented with two questions. First, they were asked “How anxious/concerned would you be that [situation described in scenario] occurred because of your sexual orientation?” with answer categories ranging from 1 = *Very unconcerned* to 6 = *Very concerned*. Second, they were asked “How likely is it that [situation described in scenario] occurred because of your sexual orientation?” with answer categories ranging from 1 = *Very unlikely* to 6 = *Very likely*. The specific wording for both questions reflected the scenario that was described in the accompanying item. Scores of anxious expectations were calculated for each item by multiplying the responses to the two questions, similar to other RS measures (Dyar et al., 2016; Pachankis et al., 2008). This way, scores reflected the interaction between anxiety about *and* expectations of rejection. The potential SMA-RSS items were all administered in Dutch. For this article, these items were translated to English in several steps. First, the first author (native Dutch speaker, fluent in English) translated the Dutch items to English. Then, these translations were checked by the second (native Dutch speaker, fluent in English) and third author (native English speaker). The product terms reflecting anxious expectations for each item were used in the EFA.

### Results

EFA using maximum likelihood estimation was conducted for the 29 potential SMA-RSS items (see Table 2) in plus version 8.3 (Muthén & Muthén, 2017). A geomin rotation was used in for the EFA and a MLR estimator was used, which is robust against assumption violations. First, according to the Cattell (1966) scree test, the number of eigenvalues that precedes the major drop in the scree plot is the number of factors that should be extracted (Fabrigar & Wegener, 2011). Following this rule, data indicated that two factors should be extracted. Second, the RMSEA also favored the two-factor model, as the RMSEA from the two-factor model onwards indicated acceptable fit (Fabrigar & Wegener, 2011). Third, parallel analyses with 5,000 permutated samples were conducted using the PARALLEL function in Mplus. It is recommended that factors are retained as long as the eigenvalue from the actual data are greater than the 95th percentile eigenvalues from the parallel analyses (O’Connor, 2000), which was the case for the first three eigenvalues. However, parallel analyses may suggest more factors than necessary, and these factors are identified by their significant but small factor loadings (Buja & Eyuboglu, 1992; O’Connor, 2000). Of note, the three strongest factor loadings on the third factor ranged from 0.43 to 0.47. By the cutoffs we used (see below), no item would be retained on this third factor leaving this factor uninterpretable, indicating that a two-factor model was preferred. Last, the two-factor model yielded a straightforward interpretation, and therefore we decided that a two-factor model was optimal. Results were supported in robustness tests where analyses were rerun for cisgender participants, assigned male at birth participants, and assigned female at birth participants, and excluding participants who identified as asexual, not sure, or reported a different sexual orientation, separately (see Table S1B in the online supplementary material).

**Table 2:** Results from the exploratory factor analyses (Study 2)

Factor number	Eigenvalue	RMSEA	95% *CI* RMSEA	95th percentile eigenvalues from parallel analysis
1	9.13	0.10	0.09–0.10	1.67
2	2.75	0.07	0.07–0.08	1.57
3	1.56	0.06	0.06–0.07	1.49
4	1.20	0.06	0.05–0.07	1.43
5	1.09	0.06	0.05–0.06	1.38
6	1.05	0.05	0.05–0.06	1.33
7	1.01	0.05	0.04–0.06	1.29

*RMSEA* root mean squared error approximation, *CI* confidence interval

Concerning a cutoff for selecting items to be retained, research suggests that setting the cutoff for factor loadings at 0.40 is liberal and setting the cutoff for factor loadings at 0.60 or more is conservative (Matsunaga, 2010). The cutoff for the primary factor loading was set at 0.50. However, items were only retained when their secondary factor loading was less than 0.30 to circumvent cross-loading (Matsunaga, 2010). In addition, robustness tests were conducted in the above-mentioned subgroups. Because of smaller sample sizes in these subgroup analyses, we chose a more liberal approach, setting the cutoff for the primary factor loading at 0.40 and the secondary factor loading at less than 0.30. This reduction resulted in retaining 14 items, eight on Factor 1 and six on Factor 2 (see Table 3 for retained items and factor loadings and Table S1C in the online supplementary material for a complete overview of all factor loadings). Of these 14 items, 10 were specifically developed for sexual minority adolescents and 4 items were adapted from existing RS scales. The correlation between the factors was 0.38 (*p* < .001), indicating a moderate association between the two factors.

**Table 3:** Factor loadings of retained items from Study 2 (EFA) and Study 3 (CFA)

Item	Factor loadings				
	EFA		Bifactor CFA		
	Factor 1	Factor 2	General factor	Group factor 1	Group factor 2
1. Imagine that you are walking through the hallway at school and a group of students is walking in your direction. When you pass them some of the students start to laugh.^a^	0.82	−0.07	0.33	0.61	
2. Imagine that you are instructed to work on a class assignment with a partner and no one wants to work with you.^a^	0.70	0.02	0.33	0.60	
3. Imagine that you are walking on the streets with some friends. You get the feeling that some youth are following you.^a^	0.66	−0.02	0.38	0.60	
4. Imagine that a group of classmates are whispering together. They look in your direction and then continue to talk.^a^	0.63	0.14	0.36	0.55	
5. Imagine that some of your classmates are celebrating a birthday of another classmate. You are not invited.^b^	0.61	0.05	0.37	0.43	
6. Imagine that only you and a group of macho men are on a train. They look in your direction and laugh.^b^	0.60	0.00	0.26	0.64	
7. Imagine that you are giving a presentation in class and a classmate laughs at you.^a^	0.59	−0.06	0.40	0.40	
8. Imagine that you are at work and a customer indicates they do not want to be helped by you.^a^	0.53	0.11	0.78	−0.13	
9. Imagine that you are watching a series with a LGBT character in it. One of your parents enters the room and says that there are too many gay people on TV.^a^	−0.08	0.77	0.70		0.06
10. Imagine that someone in your family makes a joke about LGBT people.^a^	−0.08	0.74	0.77		0.34
11. Imagine that you are watching TV with your parents. There is a program/show about LGBT rights on TV. They change the channel.^a^	−0.04	0.73	0.56		−0.20
12. Imagine that you are at a family celebration with your same-sex partner. You notice your relatives looking at you, but they don't come over to talk to you.^b^	−0.04	0.71	0.58		−0.37
13. Imagine that you are on a date with someone of the same sex at a restaurant. Your waiter provides you and your date with poor service.^b^	0.17	0.54	0.44		−0.56
14 Imagine that you are walking into a shop holding hands with your same-sex partner. Other customers stare at you.^a^	0.07	0.52	0.37		−0.14

*EFA* exploratory factor analysis, *CFA* confirmatory factor analysis

^a^These items were specifically developed for sexual minority adolescents

^b^These items were adapted from existing sexual orientation-related rejection sensitivity scales

Items that loaded on the first factor described scenarios in which someone was treated differently or excluded by others without an explicit cue that this might be related to one’s sexual orientation (e.g., a situation where someone laughs when they pass you) and perpetrators were more often peers. Items that loaded on the second factor described scenarios in which there was a more explicit cue that sexual orientation might play a role in whether someone would be excluded or treated differently (e.g., a situation involving a same-sex partner or a joke about LGBT people). Here, perpetrators were more often adults.

### Discussion

In Study 2, we tested the 29 SMA-RSS items, identified the 14 best performing items, and examined the factor structure. Contrary to previous measures of sexual orientation-related RS (Dyar et al., 2016; Pachankis et al., 2008), the EFA findings indicated that a two-factor model best fit the data.

## Study 3

The aims of Study 3 were twofold: first, to conduct a confirmatory factor analysis (CFA) (Harrington, 2009) of the SMA-RSS, and second, to assess criterion validity, convergent validity, incremental validity, and the test–retest reliability of the SMA-RSS. Focusing on criterion validity, research among sexual minority adults has shown that sexual orientation-related RS is associated with adverse mental health outcomes such as depression and anxiety (Cohen et al., 2016; Dyar et al., 2016; Feinstein et al., 2017). A different indicator of poor mental health is psychosomatic complaints, which are understood as subjective physical complaints without an objective explanatory pathology (Campo, 2012) and associated with depression and anxiety among adolescents (Campo, 2012). Therefore, we expected that the SMA-RSS would be positively associated with depression, anxiety, and psychosomatic complaints. Further, research on sexual orientation-related RS shows positive associations with minority stressors such as prejudice events, concealment, and internalized stigma, as well as a negative association with disclosure (Dyar et al., 2016; Feinstein et al., 2012; Pachankis et al., 2008). Therefore, we expected that the SMA-RSS would be positively associated with prejudice events, concealment, and internalized stigma and negatively associated with disclosure. Last, general RS, understood as expectations of rejection not related to a specific identity, is often moderately to highly associated with sexual orientation-related RS (Dyar et al., 2016). Therefore, we expected that general RS would be positively associated with the SMA-RSS and this way test convergent validity.

Although we expected that the SMA-RSS would be associated with minority stressors and general RS, they should theoretically measure different constructs. To test incremental validity, we expected that the SMA-RSS would be associated with mental health outcomes above and beyond minority stressors and general RS.

Further, most research has conceptualized sexual orientation-related RS as a trait (Feinstein, 2020). However, a longitudinal study among sexual minority male college students found a significant decrease in scores on the GR-RSS over time (Pachankis et al., 2018). Therefore, we also assessed the one-month test–retest reliability of the SMA-RSS.

### Method

#### Participants

In total**,** 1,010 participants opened the survey. Of those, 2 were identified as mischievous responses (i.e., joke responses to open-ended questions), 5 were duplicate cases (e.g., the same email address was used for two participants), 53 were deemed ineligible to participate in the study based on not correctly answering three multiple-choice questions to check whether they fully understood what they were consenting to (described below), 25 did not fall in the correct age range of 14–18 years, 416 skipped all items about RS, and 10 identified as heterosexual. Data from these participants were omitted from the sample, resulting in an analytic sample of 499. This sample size is sufficient as a priori power calculations using g-power indicated that a sample size of at least 143 was needed to find medium sized effects in linear regression analyses at *p* = .05, a power of 0.80, and six predictors (Faul et al., 2007). Further, with communalities under and around 0.50 (mean in our study was 0.36), a small number of factors (under 4), and an overdetermination of factors (6 items per factor, also the minimum in our study) the sample size is considered acceptable (MacCallum et al., 1999).

#### Procedure

Procedures were identical to those as described for Study 2. The only differences were that participants were explained that they would complete a survey of approximately *20 min* and that participants were asked whether the research team could contact them to participate in a future study (to assess test–retest reliability of the SMA-RSS). The questionnaire was administered in Dutch.

#### Measures

##### SMA-RSS

Participants were asked to respond to the 14 best performing items identified in Study 2 (see Appendix 1 [English version] and the online supplementary [Dutch version] for the complete measure; the translation procedure is described in Study 2). Assessment of the SMA-RSS was similar as described in Study 2. Contrary to previous RS scales (Chan & Mendoza-Denton, 2008; Downey & Feldman, 1996; Dyar et al., 2016; London et al., 2012; Pachankis et al., 2008), we found that a two-factor solution best fit the data in Study 2. Because of this, the calculation of the total SMA-RSS measure (e.g., calculating a mean score) depended on the best fitting CFA model. The product terms reflecting anxious expectations for each item were used in the CFA.

##### Depressive Symptoms

To assess depressive symptoms, we used the major depressive disorder subscale of the Dutch Revised Child Anxiety and Depression Scale (RCADS) (Chorpita et al., 2000; Kösters et al., 2015; Mathyssek et al., 2013; Muris et al., 2002). Participants were presented with 10 items (e.g., “I feel very worthless”) and were asked how often they feel this way. Answer options were 0 = *Never*, 1 = *Sometimes*, 2 = *Often*, and 3 = *Always*. Participants received the items of the RCADS in random order. The mean score of these 10 items was calculated so that higher scores indicate more depressive symptoms (α = 0.90).

##### Anxiety Symptoms

For anxiety symptoms, we used the generalized anxiety disorder subscale of the Dutch RCADS (Chorpita et al., 2000; Kösters et al., 2015; Mathyssek et al., 2013; Muris et al., 2002). Participants were presented six items (e.g., “I worry about bad things happening to me”) and were asked how often they feel this way. Answer options were 0 = *Never*, 1 = *Sometimes*, 2 = *Often*, and 3 = *Always*. Participants received the items of the RCADS in random order. Again, the mean score was calculated, with higher scores indicating more anxiety symptoms (α = 0.83).

##### Psychosomatic Complaints

To assess psychosomatic complaints, we used the Dutch Health Behaviour in School-aged Children (HBSC) symptom checklist (Ravens-Sieberer et al., 2008). Participants were asked how often they had experienced eight psychosomatic complaints in the last six months (e.g., headache, abdominal pain) with answer options ranging from 1 = *Almost never* through 5 = *Almost every* day. The mean score of these eight items was calculated, and higher scores indicated more psychosomatic complaints (α = 0.83).

##### Prejudice Events

To assess prejudice events, participants were asked how often they experienced five forms of victimization related to their sexual identity in the past year. An example of a victimization experience is “Physical violence being used against you” (Baams et al., 2015; D’Augelli et al., 2008). This measure has been used in the Netherlands before to assess prejudice events among sexual minority youth (Baams et al., 2015). Answer options were 0 = *This did not happen*, 1 = *Once or twice per year*, 2 = *Two or three times per month*, 3 = *Almost every week*, and 4 = *A couple of times every week*. The mean score of these five items was calculated, with higher scores reflecting frequent experiences with prejudice events in the past year (α = 0.78).

##### Concealment

The Nebraska Outness Scale-Concealment was used to assess concealment (Meidlinger & Hope, 2014). Participants were asked how often they would hide their sexual identity by, for example, not talking about sexual identity-related subjects when interacting with their immediate family (e.g., parents, siblings), people they socialize with (e.g., friends), schoolmates, and coworkers. Answer options ranged from 1 = *Never* through 5 = *Always*. For each group, participants could also indicate “*does not apply*,” which was coded as missing. The mean score was calculated with higher scores reflecting higher rates of concealment (α = 0.80). Items were translated to Dutch by the first author (native Dutch speaker, fluent in English) and subsequently checked by the second author (native Dutch speaker, fluent in English).

##### Disclosure

To assess level of disclosure, the Nebraska Outness Scale-Disclosure measure was used (Meidlinger & Hope, 2014). Participants were asked what percentage of people in specific groups (i.e., immediate family, people you socialize with, schoolmates, and coworkers) were aware of participants’ sexual orientation. Participants could indicate a percentage for each group and could also indicate “*does not apply*,” which was coded as missing. The mean score was calculated with higher scores reflecting higher disclosure (α = 0.81). Items were translated to Dutch by the first author (native Dutch speaker, fluent in English) and subsequently checked by the second author (native Dutch speaker, fluent in English).

##### Internalized Stigma

For internalized stigma, participants were asked five questions about negative attitudes they had about their own sexual identity from the Lesbian Internalized Homophobia Scale (Bos et al., 2004; Szymanski et al., 2001), which has been successfully adapted and used in Dutch sexual minority youth samples (Baams et al., 2014). An example item was “I wish I did not have this sexual identity” and answer options ranged from 1 = *Totally disagree* through 5 = *Totally agree*. The mean score of these five items was calculated, with higher scores reflecting higher internalized stigma (α = 0.76).

##### General RS

Participants were asked to respond to the short 8-item General RS questionnaire (Downey & Feldman, 1996). Participants were presented with eight items in which a situation was described in which they might be rejected by a close other. An example item reads “You approach a close friend to talk after doing or saying something that seriously upset him/her.” Similar to the SMA-RSS, two specific questions (one about anxiety and one about expectations or rejection) followed each item. Scores were calculated for each item by multiplying the responses to the two questions and calculating the mean across all eight product scores (α = 0.78). Although initially not developed for adolescents, research has used the general RS questionnaire in adolescent samples successfully (Chango et al., 2012; Hafen et al., 2014; Marston et al., 2010). Items were translated to Dutch by the first author (native Dutch speaker, fluent in English) and subsequently checked by the second author (native Dutch speaker, fluent in English).

#### Analytic Strategy

First, we conducted several CFA analyses to assess what type of model best fit the structure of the SMA-RSS. First, a correlated two-factor model was estimated where items could load on two correlated factors. Then, two nested models with different structures were estimated. These nested models are often estimated to fit multidimensional data that represent different domains of one higher construct (Mansolf & Reise, 2017). First, a second-order model was estimated where items could load on orthogonal first-order factors and where the first-order factors themselves loaded on one second-order factor. Last, we estimated a bifactor model, where items were allowed to load both on one general factor and two so-called group factors where all factors are orthogonal. Although similar, second-order models and bifactor models imply different structures of the SMA-RSS and how they should be represented in structural equation modeling research. Several fit indices were assessed to evaluate model fit: the root mean square error of approximation (RMSEA), where values < 0.08 indicate good fit; the comparative fit index (CFI), where values > 0.90 indicate good fit; and the Akaike’s information criterion (AIC) and sample sized adjusted Bayesian information criterion (Adj BIC), where lower values indicate better fit (Harrington, 2009). Based on these outcomes, the calculation of the total SMA-RSS measure was determined. All analyses were conducted in Mplus version 8.3 (Muthén & Muthén, 2017).

Next, correlations were assessed between the SMA-RSS and depressive symptoms, anxiety symptoms, psychosomatic complaints, prejudice events, concealment, internalized stigma, and general RS. After that, separate linear regression analyses were estimated with depressive symptoms, anxiety symptoms, and psychosomatic complaints as dependent variables. The associations of the SMA-RSS adjusted for either prejudice events, concealment, disclosure, internalized stigma, or general RS were assessed. We also estimated a model including al predictors simultaneously. Because of this, for every dependent variable, a total of 16 significance tests were conducted. To correct for multiple testing for each outcome variable, we used the false discovery rate (FDR) method (Benjamini & Hochberg, 1995). For the FDR method, the *p* values of all significance tests for a specific dependent variable were ordered from smallest to largest. A result was considered statistically significant if for the ith-ordered *p* value *p*(i) ≤ α × i/16, where α was set at .05 and i is the ranking in the order of *p* values. Last, test–retest reliability of the SMA-RSS was assessed by inspecting the intraclass correlation (ICC). Several robustness checks were conducted for the following subgroups: cisgender participants, assigned male at birth participants, assigned female at birth participants, and excluding participants who identified as asexual, not sure, or with a different sexual orientation. For all models, a MLR estimator was used, which is robust to model assumption violations.

### Results

Table 4 presents model fit indices for the estimated CFA models. The bifactor model had the lowest RMSEA and highest CFI, which both indicated acceptable fit. The AIC and the adjusted BIC were smallest for the bifactor model. Taken together, several fit indices pointed to the bifactor model. Robustness checks confirmed that the bifactor had the best fit as well (see Table S1D in the online supplementary).

**Table 4:** Model fit of several confirmatory factor analyses (Study 3)

	RMSEA	90% *CI* RMSEA	CFI	AIC	Adj BIC
Correlated 2-factor model	0.10	09–0.10	0.75	43,566.74	46,611.40
Second-order factor model	0.10	0.09–0.10	0.74	43,568.74	43,614.44
Bifactor model	0.06	0.05–0.07	0.91	43,258.86	43,317.02

*RMSEA* root mean squared error approximation, *CI* confidence interval, *CFI* comparative fit index, *Adj BIC* adjusted Bayesian information criterion

Despite finding two factors (i.e., multidimensionality) for the SMA-RSS, we examined whether the general factor was strong enough to justify unidimensionality. Further, we assessed the model-based reliability by studying how much score variance was explained by the general factor and the group factors and whether the general and group factors measure the construct(s) of interest. Additionally, we assessed whether the total score is a sufficiently reliable measure of the general factor (Reise et al., 2013; Rodriguez et al., 2016).

The unidimensionality of the SMA-RSS was assessed by checking the explained common variance (ECV), percent uncontaminated correlations (PUC), and Omega Hierarchical (ωH). The ECV reflects the proportion of common variance across the items explained by the general factor. The PUC is the percentage of correlations that directly inform the general factor. The ωH reflects the proportion in the total score variance that can be attributed to the general factor while accounting for group factors (Rodriguez et al., 2016). It has been suggested that when PUC < 0.80, ECV > 0.60, and ωH > 0.70, an instrument can be qualified as primarily unidimensional (Reise et al., 2013). In our study, the PUC was 0.53, the ECV was 0.66, and the ωH was 0.74, which would suggest unidimensionality. This was also supported in robustness checks (see Table S1E in the online supplementary material). Then, the item loadings on the group and the general factors were inspected, where larger loadings on the general than the group factors indicate that the items better measure the general factor. Six item loadings were larger for the general factor, one was equal, and seven were larger for the group factors (see Table 3), meaning that the factor loadings partially supported unidimensionality. Robustness checks supported this as well (see Table S1F in the online supplementary material). Taken together, we found partial support for unidimensionality of the SMA-RSS. Given that we found partial support for unidimensionality of the SMA-RSS and that all prior measures of RS have had a single factor, we determined that it was best to operationalize the SMA-RSS as a unidimensional measure.

The model-based reliability of the SMA-RSS was assessed by inspecting the Omega (ω), Omega Subscale (ωS), Omega Hierarchical (ωH), and Omega Hierarchical Subscale (ωHS) (Rodriguez et al., 2016). The ω reflects the proportion of score variance attributed to the general and group factors and was 0.91. The ωS reflects the proportion of subscale score variance attributed to the general and group factors and was 0.83 for the first and 0.80 for the second factor. The ωH reflects the proportion in the score variance that can be attributed to the general factor while accounting for group factors and was 0.74. Generally, if ωH > 0.75, the raw total score can be used to measure the general factor (Reise et al., 2013). The ωHS reflects the proportion of subscale score variance that can be attributed to the group factor while accounting for the general factor and was 0.40 for the first and 0.04 for the second group factor. A ωHS < 0.50 indicates that most subscale variance can be attributed to the general factor, which means that the two group factors do not measure unique constructs (Reise et al., 2013). Similar values were obtained in robustness checks (see Table S1E in the online supplementary material). Again, based on these findings and to stay consistent with previous RS measures, the mean score of the SMA-RSS was used in subsequent analyses. Of note, we present results of sensitivity analyses with the two separate group factors in the online supplement.

#### Criterion Validity

Correlations between the SMA-RSS and depressive symptoms, anxiety symptoms, psychosomatic complaints, prejudice events, concealment, disclosure, and internalized stigma were examined (see Table 5). As expected, higher scores on the SMA-RSS were associated with higher levels of depressive symptoms and psychosomatic complaints (small to moderate effects) and higher levels of anxiety symptoms (a moderate effect). Further, we found small to moderate positive correlations with concealment and internalized stigma and a moderate positive correlation with prejudice events, all consistent with our expectations. Contrary to our expectations, no significant association was found with disclosure, although the direction of the association was, as expected, negative. Among participants assigned male at birth, correlations between the SMA-RSS and depressive symptoms and psychosomatic complaints were not significant (see Table S1G and S1H in the online supplementary material). The same pattern of results was found when the two group factors of the SMA-RSS were assessed separately (see Table S1I in the online supplementary material).

**Table 5:** Descriptive statistics and correlations among key variables (Study 3)

	*M*	*SD*	Min–Max	1	2	3	4	5	6	7	8	9
1. SMA-RSS	9.39	5.23	1.00–28.14	–								
2. Depressive symptoms	2.26	0.62	1.00–4.00	**0.27**	–							
3. Anxiety symptoms	2.25	0.63	1.00–4.00	**0.37**	**0.66**	–						
4. Psychosomatic complaints	2.65	0.92	1.00–5.00	**0.29**	**0.79**	**0.62**	–					
5. Prejudice events	1.82	0.67	1.00–4.60	**0.43**	**0.40**	**0.35**	**0.44**	–				
6. Concealment	2.88	1.07	1.00–5.00	**0.30**	**0.22**	**0.15**	**0.21**	0.07	–			
7. Disclosure	51.28	29.18	0.00–100.00	−0.07	−0.05	0.00	0.00	**0.16**	**−0.59**	–		
8. Internalized stigma	2.04	0.77	1.00–4.60	**0.32**	**0.24**	**0.21**	**0.21**	**0.12**	**0.41**	**−0.29**	–	
9. General RS	6.89	3.96	1.00–25.75	**0.38**	**0.36**	**0.39**	**0.36**	**0.27**	**0.24**	**−0.10**	**0.18**	–

*M* mean, *SD* standard deviation, *SMA-RSS* Sexual Minority Adolescent Rejection Sensitivity Scale, *RS* rejection sensitivity

Bold numbers indicate *p* < .05

#### Convergent Validity

Correlations between the SMA-RSS and general RS were assessed (see Table 5). Higher scores on the SMA-RSS were associated with higher general RS (a moderate effect). Similar correlations were found in robustness checks (see Table S1G till S1I) in the online supplementary material.

#### Incremental Validity

Table 6 displays the standardized regression coefficients. The results indicated that the SMA-RSS was generally associated with more depressive symptoms, anxiety symptoms, and psychosomatic complaints above and beyond prejudice events, concealment, disclosure, internalized stigma, and general RS. In models including all predictors simultaneously, the SMA-RSS was only associated with more anxiety symptoms. Our findings were generally supported in robustness checks (see Table S1J in the online supplementary material). However, for assigned male at birth participants, the SMA-RSS was generally not significantly associated with depressive symptoms, anxiety symptoms, and psychosomatic complaints above and beyond minority stressors and general RS. In analyses where the two factors of the SMA-RSS were assessed separately, the first factor was associated with anxiety symptoms over and beyond concealment, disclosure, internalized stigma, and general RS. The second factor was associated with depressive symptoms, anxiety symptoms, and psychosomatic complaints above and beyond prejudice events, concealment, disclosure, and internalized stigma (see Table S1K in the online supplementary material). That less significant associations were found for the first factor is likely related to confounding of the second factor.

**Table 6:** Regression analyses with the SMA-RSS predicting depressive symptoms, anxiety symptoms, and psychosomatic complaints above and beyond minority stressors and general rejection sensitivity (Study 3)

	Depressive symptoms		Anxiety symptoms		Psychosomatic complaints	
	ß	SE	ß	SE	ß	SE
Prejudice events	**0.35**	0.05	**0.24**	0.05	**0.39**	0.05
SMA-RSS	**0.12**	0.06	**0.27**	0.06	**0.13**	0.05
Concealment	**0.17**	0.05	0.06	0.05	**0.12**	0.06
SMA-RSS	**0.22**	0.06	**0.35**	0.05	**0.26**	0.06
Disclosure	−0.04	0.05	0.03	0.05	0.03	0.05
SMA-RSS	**0.26**	0.05	**0.37**	0.05	**0.29**	0.05
Internalized stigma	**0.17**	0.05	0.10	0.05	**0.12**	0.05
SMA-RSS	**0.21**	0.05	**0.34**	0.05	**0.26**	0.05
General RS	**0.31**	0.05	**0.30**	0.05	**0.30**	0.05
SMA-RSS	**0.16**	0.06	**0.27**	0.06	**0.18**	0.06
Prejudice events	**0.33**	0.05	**0.21**	0.06	**0.36**	0.05
Concealment	**0.14**	0.06	0.04	0.07	**0.14**	0.06
Disclosure	0.03	0.06	0.06	0.06	0.08	0.06
Internalized stigma	**0.13**	0.06	0.10	0.06	0.09	0.06
General RS	**0.25**	0.05	**0.27**	0.05	**0.24**	0.05
SMA-RSS	−0.03	0.06	**0.16**	0.06	−0.01	0.06

*SMA-RSS* Sexual Minority Adolescent Rejection Sensitivity Scale, *RS* rejection sensitivity

Bold numbers indicate *p* < .05

#### Test–Retest Reliability

##### Participants

In total, 141 participants opened the test–retest survey. Of those, 26 skipped all items about RS and their data was omitted, resulting in an analytic sample of 115. Of these participants, 25.5% identified as gay, 20.0% as lesbian, 28.7% as bisexual, 7.8% as queer, 8.7% as pansexual, 0.9% as asexual, 2.6% were not sure, and 6.1% had a different sexual orientation than the labels presented in the survey (e.g., demisexual). In total, 33.0% were assigned male at birth and 67.0% were assigned female at birth. For gender identity, 28.7% identified as cisgender men, 32.2% as cisgender women, and 39.1% identified as a gender minority (e.g., transgender, non-binary). About 80.0% still attended either high school vocational education, or (applied) university. The mean age of the test–retest sample was 16.87 (*SD* = 1.02).

##### Procedure

In total, 253 participants of Study 3 consented to being contacted to participate in a future study and received an email invitation to fill out a short questionnaire one-month after completing the main survey of Study 3. In the email, it was explained that participants would complete a survey of approximately five minutes and that they would receive no compensation. We did not perform an extra check whether participants fully understood to what they consented because this was already done for the main survey of Study 3.

##### Measures

Participants were asked to respond to the 14 items of SMA-RSS. Procedures and scale construction of the SMA-RSS were the same as described for the main survey of Study 3.

##### Results

An ICC of 0.73 was found, which means that 73% of the variance between the first and the second assessment of the SMA-RSS can be attributed to differences between people and scores on the SMA-RSS could be categorized as moderately stable (Koo & Li, 2016). No robustness checks were conducted because of the relatively small sample size in the test–retest sample.

### Discussion

In Study 3, we conducted several CFAs for the SMA-RSS and found that the bifactor model best fit the data. We provided some evidence for the unidimensionality of the SMA-RSS and the use of the raw mean score, in line with previous RS measures. We further assessed criterion validity, convergent validity, incremental validity, and the test–retest reliability. Criterion validity was supported by the correlations between the SMA-RSS and the other measures of minority stress. Convergent validity was supported by the correlation between the SMA-RSS and general RS. The SMA-RSS was still associated with mental health outcomes when we controlled for minority stressors and general RS, providing evidence for incremental validity. For test–retest reliability, participants were found to be moderately stable in their scores on the SMA-RSS over a one-month time period.

## General Discussion

Previous research has pointed to sexual orientation-related RS as a potential mechanism through which mental health disparities among sexual minority people can be explained (Feinstein, 2020). Nevertheless, research on sexual orientation-related RS among sexual minority adolescents is scarce (Baams et al., 2020). Previous measures of RS are population-specific and were developed specifically for sexual minority adult men (Pachankis et al., 2008) and women (Dyar et al., 2016). To date, no measure of sexual orientation-related RS has been developed specifically for sexual minority adolescents. Therefore, the aim of this study was to develop and validate a measure of RS for sexual minority adolescents, the SMA-RSS.

Our interviews with sexual minority youth indicated that the social contexts in which sexual minority adolescents anxiously expect, readily perceive, or intensely react to sexual orientation-based rejection were not well represented in existing RS measures for adults. This underlined the need for a developmentally appropriate measure of RS for sexual minority adolescents. This fits with research that emphasizes that sexual minority adolescents encounter or expect stigma in different social contexts than sexual minority adults (Goldbach & Gibbs, 2017), and that measures of status-based RS require population-specific items (Feinstein, 2020).

In two separate samples (Study 2 and 3), a multidimensional factor structure of the SMA-RSS was found. However, in Study 3, the bifactor analysis provided evidence that the general factor was strong enough to justify a unidimensional model and that the total test score of the SMA-RSS was sufficiently reliable to use. However, it should be noted that some of the statistics used to assess unidimensionality were close to the cutoff scores and future research should therefore aim to further study the unidimensionality of the scale. Considering that previous RS measures had a unidimensional factor structure (Downey & Feldman, 1996; Dyar et al., 2016; London et al., 2012; Mendoza-Denton et al., 2002; Pachankis et al., 2008), it is noteworthy that the SMA-RSS showed a multidimensional factor structure. A possible source of this multidimensionality could be found in the contents of the scenarios in the SMA-RSS. That is, some items described scenarios in which someone was treated differently or excluded by others without an explicit cue that this might be related to one’s sexual orientation and perpetrators were more often adolescents or youth. In other scenarios, there was a more explicit cue that sexual orientation might play a role in whether someone would be excluded or treated differently and perpetrator were more often adults. That the multidimensionality could result from differences in the wording of the questions is described as a potential source of multidimensionality in bifactor models (Wang et al., 2018).

Further, we demonstrated evidence for the criterion validity of the SMA-RSS. As expected, the SMA-RSS was associated higher levels of minority stress, except for disclosure. This is consistent with knowledge of sexual orientation-related RS among adults (Dyar et al., 2016; Feinstein et al., 2012; Pachankis et al., 2008). Although we expected that the SMA-RSS would be negatively associated with disclosure, we found no significant association. This finding suggests that regardless of the extent to which someone discloses their sexual orientation to others, sexual minority adolescents might still anxiously expect rejection in certain situations. With regard to mental health outcomes, the SMA-RSS was associated with higher levels of mental health problems, which is consistent with previous research on sexual orientation-related RS among adults (Dyar et al., 2016; Pachankis et al., 2008).

Evidence for convergent validity was found as well. The SMA-RSS was positively associated with general RS. This underlines that general feelings of RS and sexual orientation-related RS are related but different constructs and demonstrates the relevancy of population-specific RS measures.

Evidence for incremental validity was found as well. The SMA-RSS was associated with higher levels of mental health problems above and beyond prejudice events, concealment, disclosure, and internalized stigma. In models containing all predictors, the SMA-RSS was only associated with higher levels of anxiety symptoms. Given that these models included 6 predictors, it is possible that there was limited unique variance for the SMA-RSS to account for when all of these predictors were included in the same model. Overall, these findings are especially relevant as literature often uses the minority stress framework to explain poorer mental health outcomes among sexual minority adolescents (Dürrbaum & Sattler, 2020). We demonstrated that the SMA-RSS can potentially explain adverse health outcomes among sexual minority adolescents above and beyond these often-studied mechanisms, which could further our understanding of health disparities among sexual minority youth. We also demonstrated that the SMA-RSS was associated with mental health above and beyond general RS. Past research has noted that identity-based RS measures should be able to outperform general RS measures when predicting health outcomes (Dyar et al., 2016), which the SMA-RSS was able to do. This strengthens evidence for the unique predictive power of the SMA-RSS in predicting mental health among sexual minority adolescents.

Last, we demonstrated test–retest reliability of the SMA-RSS: Over a one-month period, sexual minority adolescents were moderately stable in their feelings of sexual orientation-related RS (Koo & Li, 2016), which is consistent with the view of RS being a trait (Feinstein, 2020). Although a longitudinal study among gay and bisexual men found a decrease in sexual orientation-related RS over an eight-year time period (Pachankis et al., 2018), they assessed RS on an annual basis. As such, it is possible that sexual orientation-related RS is stable over shorter periods of time (e.g., one-month) but fluctuates over longer periods of time (e.g., one-year). Future studies with more measurements of the SMA-RSS are therefore needed.

Taken together, the SMA-RSS captures unique situations in which sexual minority adolescents anxiously expect, readily perceive, and intensely react to sexual orientation-based rejection. We demonstrated how the SMA-RSS is distinct from known minority stressors and general RS and can predict mental health outcomes even when controlling for minority stressors and general RS. Similar to studies among sexual minority adults (Cohen et al., 2016; Feinstein et al., 2012), the development of the SMA-RSS provides researchers with the opportunity to examine the direct association between sexual orientation-related RS and mental health and how it might explain associations between distal minority stressors and mental health outcomes. Ultimately, this type of research will improve our understanding of how disparities in mental health might develop among sexual minority adolescents. Practitioners should be aware that sexual minority adolescents anxiously expect rejection in unique situations compared with sexual minority adults. They should also be cognizant that mere anxious expectation of rejection based on their sexual orientation might already be taxing for adolescents’ mental health and that reducing these feelings could improve their wellbeing.

### Limitations and Future Directions

Our findings should be interpreted in light of some limitations. First, participants in all studies were recruited using advertisements on Facebook and Instagram that targeted youth with sexual minority-related interests. This affects the generalizability of the current findings. Youth who have sexual minority-related interests on social media may have lower levels of sexual orientation-related RS, because they do not anxiously expect rejection based on expressing these interests. Second, correlational data was used in all studies and therefore we cannot make inferences about the causality of associations. A longitudinal design might give some indication of temporal development of sexual orientation-related RS and mental health. Third, we designed the SMA-RSS to be used with all sexual minority adolescents, regardless of sex, gender, or specific sexual orientation. A potential drawback of this approach is that it might not reflect scenarios in which subgroups of sexual minority adolescents (e.g., boys vs. girls) respond differently to sexual orientation-based rejection. However, the benefit of creating a broad measure is that it can be used in research with a diverse group of sexual minority adolescents. Last, sexual orientation-related RS is context dependent and the present study was conducted in the Netherlands. It could be that some of the items are less relevant in other cultural contexts or countries, although in developing the items we took care in making them broadly applicable.

Future research should also focus on how sexual orientation-related RS develops and what important developmental periods are. Considering that disparities in victimization between sexual minority and heterosexual children have been observed already at age 9 (Martin-Storey & Fish, 2019; Mittleman, 2019), we might expect that sexual orientation RS also emerges at a young age (Baams et al., 2020) and further develops during adolescence and later life. If so, this would call for early interventions in, for instance, bias-based bullying or other forms of minority stress.

### Conclusion

We have developed a measure for sexual orientation-related RS among sexual minority adolescents and provided support for the reliability and validity of the measure. Future research on health disparities among sexual minority adolescents is encouraged to consider the role of sexual orientation-related RS as evidence suggests it play an important role in explaining poorer mental health in this population.

### Electronic supplementary material

Below is the link to the electronic supplementary material.

Supplementary file1 (DOCX 112 KB)

## Data Availability

Data of Studies 1–2 are not available. Final versions of the SMA-RSS are available in English (see Appendix 1) and Dutch (see online supplementary).

## Appendix 1

The Sexual Minority Adolescent Rejection Sensitivity Scale (SMA-RSS)

**Table:**

Scoring procedure
To obtain a score for each participant you have to:
1. Multiply the answer to question 1 and question 2 for each scenario
2. Add the product scores of the 14 questions and subsequently divide by 14

**Introductory text**
We are now going to present you with several situations. They all start with 'Imagine that…'. Try to imagine as best as possible what this situation would be like for you. After every situation, there are two questions. Some of the situations are about a same-sex partner; you can also read this as a same-gender partner

**Scenario 1. Imagine that you are walking through the hallway at school and a group of students is walking in your direction. When you pass them some of the students start to laugh**						
Question 1. How concerned or anxious would you be that they are laughing because of your sexual orientation?	Very unconcerned			Very concerned		
	1	2	3	4	5	6
Question 2. How likely is it that they are laughing because of your sexual orientation?	Very unlikely			Very likely		
	1	2	3	4	5	6

**Scenario 2. Imagine that you are instructed to work on a class assignment with a partner and no one wants to work with you**						
Question 1. How concerned or anxious would you be that no one wants to work with you because of your sexual orientation?	Very unconcerned			Very concerned		
	1	2	3	4	5	6
Question 2. How likely is it that no one wants to work with you because of your sexual orientation?	Very unlikely			Very likely		
	1	2	3	4	5	6

**Scenario 3. Imagine that you are walking on the streets with some friends. You get the feeling that some youth are following you**						
Question 1. How concerned or anxious would you be that you are being followed because of your sexual orientation?	Very unconcerned			Very concerned		
	1	2	3	4	5	6
Question 2. How likely is it that you are being followed because of your sexual orientation?	Very unlikely			Very likely		
	1	2	3	4	5	6

**Scenario 4. Imagine that a group of classmates are whispering together. They look in your direction and then continue to talk**						
Question 1. How concerned or anxious would you be that they are whispering about you because of your sexual orientation?	Very unconcerned			Very concerned		
	1	2	3	4	5	6
Question 2. How likely is it that they are whispering about you because of your sexual orientation?	Very unlikely			Very likely		
	1	2	3	4	5	6

**Scenario 5. Imagine that some of your classmates are celebrating a birthday of another classmate. You are not invited**						
Question 1. How concerned or anxious would you be that you are not invited because of your sexual orientation?	Very unconcerned			Very concerned		
	1	2	3	4	5	6
Question 2. How likely is it that you are not invited because of your sexual orientation?	Very unlikely			Very likely		
	1	2	3	4	5	6

**Scenario 6. Imagine that only you and a group of macho men are on a train. They look in your direction and laugh**						
Question 1. How concerned or anxious would you be that they are laughing because of your sexual orientation?	Very unconcerned			Very concerned		
	1	2	3	4	5	6
Question 2. How likely is it that that they are laughing because of your sexual orientation?	Very unlikely			Very likely		
	1	2	3	4	5	6

**Scenario 7. Imagine that you are giving a presentation in class and a classmate laughs at you**						
Question 1. How concerned or anxious would you be that they are laughing because of your sexual orientation?	Very unconcerned			Very concerned		
	1	2	3	4	5	6
Question 2. How likely is it that they are laughing because of your sexual orientation?	Very unlikely			Very likely		
	1	2	3	4	5	6

**Scenario 8. Imagine that you are at work and a customer indicates they do not want to be helped by you**						
Question 1. How concerned or anxious would you be that the customer does not want to be helped by you because of your sexual orientation?	Very unconcerned			Very concerned		
	1	2	3	4	5	6
Question 2. How likely is it that the customer does not want to be helped by you because of your sexual orientation?	Very unlikely			Very likely		
	1	2	3	4	5	6

**Scenario 9. Imagine that you are watching a series with a LGBT character in it. One of your parents enters the room and says that there are too many gay people on TV**						
Question 1. How concerned or anxious would you be that they would accept you less because of your sexual orientation?	Very unconcerned			Very concerned		
	1	2	3	4	5	6
Question 2. How likely is it that they would accept you less because of your sexual orientation?	Very unlikely			Very likely		
	1	2	3	4	5	6

**Scenario 10. Imagine that someone in your family makes a joke about LGBT people**						
Question 1. How concerned or anxious would you be that this person will not accept you because of your sexual orientation?	Very unconcerned			Very concerned		
	1	2	3	4	5	6
Question 2. How likely is it that this person will not accept you because of your sexual orientation?	Very unlikely			Very likely		
	1	2	3	4	5	6

**Scenario 11. Imagine that you are watching TV with your parents. There is a program/show about LGBT rights on TV. They change the channel**						
Question 1. How concerned or anxious would you be that they would accept you less because of your sexual orientation?	Very unconcerned			Very concerned		
	1	2	3	4	5	6
Question 2. How likely is it that they would accept you less because of your sexual orientation?	Very unlikely			Very likely		
	1	2	3	4	5	6

**Scenario 12. Imagine that you are at a family celebration with your same-sex partner. You notice your relatives looking at you, but they don't come over to talk to you**						
Question 1. How concerned or anxious would you be that they don’t come over to talk because of your sexual orientation?	Very unconcerned			Very concerned		
	1	2	3	4	5	6
Question 2. How likely is it that they don’t come over to talk because of your sexual orientation?	Very unlikely			Very likely		
	1	2	3	4	5	6

**Scenario 13. Imagine that you are on a date with someone of the same sex at a restaurant. Your waiter provides you and your date with poor service**						
Question 1. How concerned or anxious would you be that the waiter is being rude because of your sexual orientation?	Very unconcerned			Very concerned		
	1	2	3	4	5	6
Question 2. How likely is it that the waiter is being rude because of your sexual orientation?	Very unlikely			Very likely		
	1	2	3	4	5	6

**Scenario 14. Imagine that you are walking into a shop holding hands with your same-sex partner. Other customers stare at you**						
Question 1. How concerned or anxious would you be that they are staring at you because of your sexual orientation?	Very unconcerned			Very concerned		
	1	2	3	4	5	6
Question 2. How likely is it that they are staring at you because of your sexual orientation?	Very unlikely			Very likely		
	1	2	3	4	5	6

## Code Availability

Codes are available upon request.
