# Supplementary material for: Development and Validation of the Sexual Minority Adolescent Rejection Sensitivity Scale
Source: Arch Sex Behav. 2022 Dec 1;52(3):971–89. doi: 10.1007/s10508-022-02474-6 (PMC10102110; doi:10.1007/s10508-022-02474-6)
Supplement: Supplementary file 1 — Supplementary file1 (DOCX 112 KB) [file 10508_2022_2474_MOESM1_ESM.docx]

| Table S1A  *Overview of the Items From the GR-RSS and the SMW-RSS and Adaptations for the Current Study.* | | | | | | | |
| --- | --- | --- | --- | --- | --- | --- | --- |
| Item number | Original Items | Item used for interview? | Adjustments that were made | Reaction of participants to the item | Retained after interviews? | Adjustments made after interviews | Final item |
| SMW-RSS (Dyar et al., 2016) | | | | | | | |
| 1 | You and your female partner are having dinner together at a restaurant. A male customer approaches your table. | Yes. | "female partner" was changed to "same-sex partner.” | Participants reacted confused by this item. As if there was more to come. Further, some indicated that dating was not something they really did. | No. Because participants were confused by this item, it was dropped. Thus, this was deemed inappropriate for adolescents. |  |  |
| 2 | You and your female partner are leaving a store together holding hands. A car drives by and the driver honks the horn loudly several times | Yes. | "female partner" was changed to "same-sex partner.” | Participants could imagine that this would happen. There was variation how they explained why this would happen. | Yes. | Some slight wording changes. | Imagine that you and your same-sex partner are leaving a store together holding hands. A car drives by and the driver honks the horn loudly several times. |
| 3 | You are on a date with a woman at a restaurant. Your waiter provides you and your date with poor service. | Yes. | "date with a woman" was changed to "date with someone of the same sex". | Participants were able to imagine that this situation would happen. | Yes. | Some slight wording changes. | Imagine that you are on a date with someone of the same sex at a restaurant. Your waiter provides you and your date with poor service. |
| 4 | You and your female partner are walking together holding hands. Several men are gathered on a corner outside a bar. | Yes. | "female partner" was changed to "same-sex partner.” | At first participants reacted confused by this item. After asking what they would do in the situation or how they felt participants made clear they relate to the situation. | Yes. | Some slight wording changes. | Imagine that you and your partner of the same sex are walking together holding hands. Several men are gathered on a corner outside a bar. |
| 5 | You are at a bar with a female friend and an intoxicated male approaches you and attempts to pick you up. You turn him down and he reacts angrily, calling you a ‘‘dyke.’’ | Yes. | "female friend was changed to "same-sex friend". "calling you a ‘‘dyke.’’ was changed to "calls you names". The part about “tries to pick you up” was removed. | This was an item participants could imagine well. There was variability as to why they thought it would happen. | No. After discussion, this item was deemed too explicit for an adolescent sample. Thus, this was deemed inappropriate for adolescents. |  |  |
| 6 | You and your female partner are looking to buy a house. After looking at a house together with the realtor, the realtor fails to schedule an appointment to view another house that she represents. | No. Because adolescents typically do not buy houses. |  |  |  |  |  |
| 7 | A few of your female coworkers regularly try to set you up with men but they never try to set you up with women. | Yes. | "female coworkers " was changed to “male/female classmates”. "set you up with men" was changed to "set you up with women/men". "set you up with women" was changed to "set you up with men/women". | Participants who were attracted to multiple genders/sexes reacted differently to this item than participants who were not attracted to multiple sexes/genders. But it was easy to imagine for everyone. | Yes. | “male/female classmates” was changed into “classmates”. "set you up with men/women" was changed into "set you up with people of another sex." Thus, only some slight wording changes were made. | Imagine that a few of your classmates regularly try to set you up with people of another sex but they never try to set you up with people of the same sex. |
| 8 | You are hanging out with a group of heterosexual female coworkers, and the subject turns to boyfriends and husbands. | Yes. | "heterosexual female coworkers" was changed to "a group of male/female classmates". "turns to boyfriends and husbands" was changed to "turns to girlfriends/boyfriends.” | Similar to item 7, there were different reaction based on participants attraction. | Yes. | “a group of male/female classmates" was changed to "a group of classmates". "girlfriends/boyfriends" was changed to "dating people of the same sex ". Thus, only some slight wording changes were made. | Imagine that you are hanging out with a group of classmates, and the subject turns dating people of the same sex. |
| 9 | A new female friend of yours makes negative remarks about lesbians | Yes. | "female friend" was changed to "male/female friend”. "about lesbians" was changed to about LGB people". | This was something most participants could imagine or had experienced. There was variability if participants related this to their sexual identity or not. | Yes. | “male/female friend” was "changed to "someone". “LGB” was changed to “LGBT”. Thus, only some slight wording changes were made. | Imagine that a new friend of yours makes negative remarks about LGBT people. |
| 10 | You disclose your sexual orientation to a new friend. Your friend doesn’t express concern about it, but your friendship soon drifts apart. | Yes. |  | This prompted some mixed reactions. However, some participants described similar experiences. | Yes. | No adjustments were made. | Imagine that you disclose your sexual orientation to a new friend. Your friend doesn’t express concern about it, but your friendship soon drifts apart. |
| 11 | You walk into the locker room at the gym and begin to change. A woman near you moves to a different part of the locker room. | Yes. | "A woman near you" was changed to "someone near you". | Participants could imagine this would happen, but the gym was not a context everybody would go to. | Yes. | This item was changed to take place at a sports club. This item was thus substantially changed. | Imagine that you are changing at a sports club. A clubmate near you moves to a different part of the locker room. |
| 12 | Your supervisor begins raising concerns about your performance at work for the first time after you bring your female partner to a company picnic. | No. Because having a company picnic is not typical for jobs adolescents have. |  |  |  |  |  |
| 13 | You notice your relatives looking at you and your female partner at a family reunion, but they don’t come over to talk to you. | Yes. | "female partner" was changed to "same-sex" partner. | There were mixed reactions to this item. Some could imagine this and link it to their sexual identity, other could imagine this, but did not link it to their sexual identity. | Yes. | Some slight wording changes. | Imagine that you are at a family celebration with your same-sex partner. You notice your relatives looking at you but they don’t come over to talk to you. |
| 14 | You and your female partner are getting married. Several of your coworkers do not come to the wedding ceremony. | No. Because adolescents typically do not marry. |  |  |  |  |  |
| 15 | The principal at your child’s elementary school has never spoken to you and your partner at school events, but you often see him speaking with other parents. | No. Because adolescents typically do not have children. |  |  |  |  |  |
| 16 | During a lecture on sexual orientation, your professor includes several stereotypes about lesbian and bisexual women as if they were facts. After the lecture, you approach the professor and politely point out the incorrect aspects of the lecture. You receive a lower grade than you expected in the class. | Yes. | "your professor“ was changed to "your teacher". "stereotypes about lesbian and bisexual women" was changed to "stereotypes about LGB people". | Participants could imagine that a teacher would say something negative about LGB people, but for most it was hard to imagine that they would receive a lower grade. | Yes. | This item was substantially changed to only reflect that a teacher mentions stereotypes as if they are facts. | Imagine that during a class your teacher includes several stereotypes about LGBT people as if they were facts. |
| Pachankis et al., 2008 | | | | | | | |
| 1 | You bring a male partner to a family reunion. Two of your old-fashioned aunts don’t come talk to you even though they see you. | No. Because of overlap with item 13 by Dyar. |  |  |  |  |  |
| 2 | A 3-year old child of a distant relative is crawling on your lap. His mom comes to take him away. | Yes. | No adjustments were made. | Participants could imagine this, but hardly anyone linked this to their sexual identity. | No. Because almost none of the participants linked this to their sexual identity. Thus, this was deemed inappropriate for adolescents. |  |  |
| 3 | You’ve been dating someone for a few years now, and you receive a wedding invitation to a straight friend’s wedding. The invite was addressed only to you, not you and a guest. | No. Because typically adolescents do not go to weddings of friends. |  |  |  |  |  |
| 4 | You go to a job interview and the interviewer asks if you are married. You say that you and your partner have been together for 5 years. You later find out that you don’t get the job. | Yes. | "if you are married" was changed to "if you are in a relationship". "For 5 years" was changed to "for a while". | Participants could imagine this would happen, but there was variability in whether they thought this would happen because of their sexual identity. | Yes. | Some slight wording changes. | Imagine that you go to a job interview and the interviewer asks if you are in a relationship. You say that you have had a same-sex partner for a while now. You later find out that you don’t get the job. |
| 5 | You are going to have surgery, and the doctor tells you that he would like to give you an HIV test. | No. Because this was too specific to males. |  |  |  |  |  |
| 6 | You go to donate blood and the person who is supposed to draw your blood turns to her co-worker and says, “Why don’t you take this one?” | No. Because adolescents typically don’t donate blood. |  |  |  |  |  |
| 7 | You go get an STD check-up, and the man taking your sexual history is rude towards you. | Yes. | No adjustments were made. | Some participants indicated that they never had an STD test, while others could imagine this. | No. Because STD tests are not common among adolescents, this was dropped. Thus, this was deemed inappropriate for adolescents. |  |  |
| 8 | You bring a guy you are dating to a fancy restaurant of straight patrons, and you are seated away from everyone else in a back corner of the restaurant. | No. Because adolescents typically do not go to fancy restaurants. |  |  |  |  |  |
| 9 | Only you and a group of macho men are on a subway train late at night. They look in your direction and laugh. | Yes. | "subway" was changed in to "train" as trains are more common across the world. | Most participants could imagine or had experienced this. However, one indicated they never used a train at night. | Yes. | The part about late at night was omitted from this item. This item was thus substantially changed. | Imagine that only you and a group of macho men are on a train. They look in your direction and laugh. |
| 10 | You and your partner are on a road trip and decide to check into a hotel in a rural town. The sign out front says there are vacancies. The two of you go inside, and the woman at the front desk says that there are no rooms left. | Yes. | "You and your partner " was changed to "you and your same-sex partner". | Most participants could imagine this, but did not themselves go on road trips. | No. Because participants did no go on road trips themselves. Thus, this was deemed inappropriate for adolescents. |  |  |
| 11 | You go to a party and you and your partner are the only gay people there. No one seems interested in talking to you. | Yes. | "you and your partner are the only gay people there" was changed to "you and your same-sex partner are the only LGB people there". | Participants could imagine this would happen and there was variability in whether they would link this to their sexual identity. | Yes. | The part about being the only LGB people at the party was removed, as this cannot always be observed. This item was thus substantially changed. | Imagine that you go to a party with your same-sex partner. No one seems interested in talking to you. |
| 12 | You are in a locker room in a straight gym. One guy nearby moves to another area to change clothes. | No because of the overlap with item 11 by Dyar. |  |  |  |  |  |
| 13 | Some straight colleagues are talking about baseball. You force yourself to join the conversation, and they dismiss your input. | Yes. | "Some straight colleagues" was changed to "some straight classmates" . "baseball." was changed into "football (soccer)". | Women did not relate this to their sexual identity, but always to gender/sex. | No. Because this was not relatable to women. Thus, this was deemed inappropriate. |  |  |
| 14 | Your colleagues are celebrating a co-worker’s birthday at a restaurant. You are not invited. | Yes. | "colleagues are celebrating a co-worker’s birthday at a restaurant" was changed in to "classmates are celebrating the birthday of another classmate". | Participants could imagine this would happen, but there was variability in whether they thought this was related to their sexual identity. | Yes. | Some slight wording changes. | Imagine that some of your classmates are celebrating a birthday of another classmate. You are not invited. |

| Table S1B  *Results from the Exploratory Factor Analysis for Cisgender Participants, Assigned Male at Birth Participants, Assigned Female at Birth Participants, and* *Excluding Asexual, Not Sure, and Participants with a Different Sexual Orientation* | | | | |
| --- | --- | --- | --- | --- |
| Factor number | Eigenvalue | RMSEA | 95% *CI* RMSEA | 95th percentile Eigenvalues from parallel analysis |
|  | Cisgender | | | |
| 1 | 8.90 | .08 | .08 – .09 | 1.81 |
| 2 | 2.72 | .06 | .06 – .07 | 1.68 |
| 3 | 1.63 | .06 | .05 – .07 | 1.59 |
| 4 | 1.31 | .06 | .05 – .07 | 1.51 |
| 5 | 1.14 | .05 | .05 – .06 | 1.45 |
| 6 | 1.11 | .07 | .06 – .07 | 1.39 |
| 7 | 1.00 | .06 | .05 – .06 | 1.34 |
|  | Assigned male at birth | | | |
| 1 | 8.64 | .08 | .08 – .09 | 2.27 |
| 2 | 2.18 | .08 | .07 – .09 | 2.05 |
| 3 | 1.95 | .08 | .06 – .09 | 1.90 |
| 4 | 1.51 | .07 | .06 – .08 | 1.78 |
| 5 | 1.32 | .07 | .06 – .08 | 1.67 |
| 6 | 1.23 | .09 | .08 – .10 | 1.58 |
| 7 | 1.18 | .09 | .07 – .10 | 1.49 |
|  | Assigned female at birth | | | |
| 1 | 9.41 | .09 | .08 – .09 | 1.84 |
| 2 | 2.87 | .07 | .06 – .07 | 1.71 |
| 3 | 1.55 | .06 | .05 – .07 | 1.61 |
| 4 | 1.25 | .06 | .06 – .07 | 1.53 |
| 5 | 1.20 | .06 | .05 – .07 | 1.47 |
| 6 | 1.03 | .07 | .06 – .08 | 1.41 |
| 7 | 0.99 | .08 | .07 – .08 | 1.35 |
|  | Excluding asexual, not sure, and participants with a different sexual orientation | | | |
| 1 | 8.72 | .08 | .07 – .08 | 1.74 |
| 2 | 2.71 | .06 | .05 – .07 | 1.62 |
| 3 | 1.60 | .06 | .05 – .06 | 1.54 |
| 4 | 1.27 | .05 | .05 – .06 | 1.47 |
| 5 | 1.17 | .05 | .04 – .06 | 1.41 |
| 6 | 1.10 | .05 | .04 – .06 | 1.36 |
| 7 | 1.00 | .06 | .05 – .06 | 1.31 |
| RMSEA = root mean squared error approximation. CI = Confidence interval | | | | |

| Table S1C  *Eigenvalues for all items for the Two-Factor Model for the Complete Sample, Cisgender Participants, Assigned Male at Birth Participants, Assigned Female at Birth Participants, and* *Excluding Asexual, Not Sure, and Participants With a Different Sexual Orientation* | | | | | | | | | | |
| --- | --- | --- | --- | --- | --- | --- | --- | --- | --- | --- |
|  | Complete sample | | Cisgender | | Assigned male at birth | | Assigned female at birth | | Excluding AND participants | |
|  | Factor 1 | Factor 2 | Factor 1 | Factor 2 | Factor 1 | Factor 2 | Factor 1 | Factor 2 | Factor 1 | Factor 2 |
| 1. Imagine that you are giving a presentation in class and a classmate laughs at you | **.59** | -.06 | **.53** | -.04 | **.55** | -.01 | **.60** | -.06 | **.52** | -.02 |
| 2. Imagine that you are at work and a customer indicates they do not want to be helped by you. | **.53** | .11 | **.50** | .12 | **.57** | .15 | **.50** | .11 | **.47** | **.13** |
| 3. Imagine that you are instructed to work on a class assignment with a partner and no one wants to work with you. | **.70** | .02 | **.69** | .02 | **.77** | -.10 | **.72** | .00 | **.62** | .06 |
| 4. Imagine that someone in your family makes a joke about LGBT people. | -.08 | **.74** | -.11 | **.77** | -.16 | **.70** | -.00 | **.69** | **-.13** | **.77** |
| 5. Imagine that people at school spread gossip about you. | **.48** | **.23** | **.38** | **.30** | **.50** | .13 | **.48** | **.28** | **.39** | **.29** |
| 6. Imagine that a new friend of yours makes negative remarks about LGBT people. | .09 | **.52** | .08 | **.49** | **.26** | **.34** | .10 | **.50** | -.01 | **.55** |
| 7. Imagine that during a class your teacher includes several stereotypes about LGBT people as if they were facts. | .06 | **.58** | -.01 | **.59** | **.24** | **.38** | .04 | **.59** | .02 | **.60** |
| 8. Imagine that a group of classmates are whispering together. They look in to your direction and then continue to talk. | **.63** | .14 | **.56** | **.23** | **.62** | -.00 | **.67** | **.15** | **.56** | **.21** |
| 9. Imagine that you are walking past a group of youth on the street. They look in to your direction. | **.64** | -.08 | **.60** | -.06 | **.27** | .18 | **.74** | -.05 | **.62** | -.09 |
| 10. Imagine that you are walking through the hallway at school and a group of students is walking in your direction. When you pass them some of the students start to laugh. | **.82** | -.07 | **.76** | -.02 | **.82** | -.03 | **.79** | -.05 | **.75** | -.01 |
| 11. Imagine that you are walking on the streets with some friends. You get the feeling that some youth are following you. | **.66** | -.02 | **.65** | -.00 | **.48** | .13 | **.68** | .02 | **.67** | .00 |
| 12. Imagine that you are changing at a sportsclub. A clubmate near you moves to a different part of the locker room. | .23 | **.40** | **.27** | **.37** | **.35** | **.28** | .14 | **.48** | **.20** | **.44** |
| 13. Imagine that you are at a family celebration with your same-sex partner. You notice your relatives looking at you but they don’t come over to talk to you. | -.04 | **.71** | -.00 | **.68** | -.00 | **.73** | -.06 | **.70** | -.10 | **.74** |
| 14. Imagine that you and your partner of the same sex are walking together holding hands. Several men are gathered on a corner outside a bar. | .16 | **.40** | **.22** | **.40** | .03 | **.34** | **.18** | **.43** | **.23** | **.36** |
| 15. Imagine that only you and a group of macho men are on a train. They look in your direction and laugh. | **.60** | .00 | **.64** | -.03 | **.42** | .22 | **.59** | .07 | **.61** | -.01 |
| 16. Imagine that a couple of friends are talking about relationships. They do not involve you in this conversation. | .20 | **.43** | **.17** | **.47** | .17 | **.49** | **.24** | **.38** | .10 | **.50** |
| 17. Imagine that you are watching TV with your parents. There is an program/show about LGBT rights on TV. They change the channel. | -.04 | **.73** | -.02 | **.69** | -.00 | **.76** | -.07 | **.72** | -.01 | **.72** |
| 18. Imagine that you go to a job interview and the interviewer asks if you are in a relationship. You say that you have a same-sex partner for a while now. You later find out that you don’t get the job. | .18 | **.42** | **.16** | **.42** | .17 | **.33** | **.21** | **.40** | .12 | **.42** |
| 19. Imagine that you disclose your sexual orientation to a new friend. Your friend doesn’t express concern about it, but your friendship soon drifts apart. | .15 | **.45** | **.18** | **.45** | **.50** | .20 | .05 | **.48** | .12 | **.46** |
| 20. Imagine that you are walking into a shop holding hands with your same-sex partner. Other customers stare at you. | .07 | **.52** | .06 | **.55** | **.22** | **.43** | -.01 | **.57** | .08 | **.52** |
| 21. Imagine that you are at a (heterosexual) bar and someone is trying to flirt with you. | **.25** | **.30** | **.31** | **.29** | .04 | **.29** | **.31** | **.32** | **.21** | **.34** |
| 22. Imagine that you and your same-sex partner are leaving a store together holding hands. A car drives by and the driver honks the horn loudly several times. | .17 | **.42** | **.21** | **.49** | **.40** | **.35** | .04 | **.46** | **.24** | **.42** |
| 23. Imagine that you are on a date with someone of the same sex at a restaurant. Your waiter provides you and your date with poor service. | .17 | **.54** | **.23** | **.53** | **.29** | **.43** | .15 | **.54** | **.14** | **.59** |
| 24. Imagine that a few of your classmates regularly try to set you up with people of the other sex but they never try to set you up with people of the same sex. | **.26** | **.23** | **.27** | **.26** | .13 | **.34** | **.35** | **.15** | **.17** | **.27** |
| 25. Imagine that your teacher asks you to answer a question. You give an answer but you do not feel that the teacher takes you seriously. | **.58** | .03 | **.52** | .06 | **.36** | .11 | **.69** | -.04 | **.50** | .05 |
| 26. Imagine that you post a picture with a pride flag in it on social media. | .12 | **.42** | .11 | **.45** | .12 | **.44** | .15 | **.36** | .12 | **.44** |
| 27. Imagine that some of your classmates are celebrating a birthday of another classmate. You are not invited. | **.61** | .05 | **.55** | .10 | **.46** | .16 | **.68** | .03 | **.51** | .12 |
| 28. Imagine that you go to a party with your same-sex partner. No one seems interested in talking to you. | .21 | **.49** | **.33** | **.37** | **.30** | **.32** | **.16** | **.55** | **.17** | **.51** |
| 29. Imagine that you are watching a series with a LGBT character in it. One of your parents enters the room and says that there are too many gay people of TV. | -.08 | **.77** | -.06 | **.73** | -.03 | **.72** | -.10 | **.78** | -.07 | **.78** |
| AND = asexual, not sure, different sexual orientation.  Bold numbers indicate *p* < .05 | | | | | | | | | | |

| Table S1D  *Model Fit of Several Confirmatory Factor Analyses for Cisgender Participants, Assigned Male at Birth Participants, Assigned Female at Birth Participants, and* *Excluding Asexual, Not Sure, and Participants With a Different Sexual Orientation* | | | | | |
| --- | --- | --- | --- | --- | --- |
|  | RMSEA | 90% *CI* RMSEA | CFI | AIC | Adj BIC |
|  | Cisgender | | | | |
| Correlated 2-factor model | .09 | .08 – .10 | .76 | 27,862.72 | 27,889.55 |
| Second-order factor model | .09 | .08 – 10 | .76 | 27,864.72 | 27,892.18 |
| Bifactor model | .06 | .05 – .07 | .92 | 27,666.53 | 27,701.48 |
|  | Assigned male at birth | | | | |
| Correlated 2-factor model | .08 | .07 – .10 | .78 | 14,909.29 | 14,908.48 |
| Second-order factor model | .08 | .07 – .10 | .79 | 14,911.29 | 14,910.46 |
| Bifactor model | .07 | .05 – .09 | .88 | 14,857.52 | 14,856.45 |
|  | Assigned female at birth | | | | |
| Correlated 2-factor model | .10 | .09 – .12 | .74 | 28,306.99 | 28,333.30 |
| Second-order factor model* | - | - | - | - | - |
| Bifactor model | .07 | .05–.08 | .91 | 28,066.33 | 28,100.60 |
|  | Excluding asexual, not sure, and participants with a different sexual orientation | | | | |
| Correlated 2-factor model | .09 | .08 – .10 | .74 | 36,807.01 | 36,844.70 |
| Second-order factor model* | - | - | - | - | - |
| Bifactor model | .06 | .05 – .07 | .91 | 36,548.12 | 36,597.19 |
| RMSEA = root mean squared error approximation. *CI* = confidence interval. CFI = comparative fit index. Adj BIC = adjusted Bayesian information criterion.  * Model did not converge. | | | | | |

| Table S1E  *Ancillary Bifactor Measures for the Complete Sample, Cisgender Participants, Assigned Male at Birth Participants, Assigned Female at Birth Participants, and* *Excluding Asexual, Not Sure, and Participants With a Different Sexual Orientation* | | | | | |
| --- | --- | --- | --- | --- | --- |
|  | Complete sample | Cisgender | Assigned male at birth | Assigned female at birth | Excluding AND participants |
| ω | .91 | .91 | .90 | .92 | .90 |
| ωS (specific factor 1) | .83 | .84 | .81 | .85 | .82 |
| ωS (specific factor 2) | .80 | .80 | .77 | .82 | .79 |
| ωH | .74 | .75 | .76 | .78 | .73 |
| ωHS (specific factor 1) | .40 | .42 | .32 | .36 | .41 |
| ωHS (specific factor 2) | .04 | .01 | .00 | .07 | .05 |
| ECV | .66 | .65 | .67 | .67 | .67 |
| PUC | .53 | .53 | .53 | .53 | .53 |
| ECV = explained common variance. PUC = percent uncontaminated correlations. AND = asexual, not sure, different sexual orientation. | | | | | |

| Table S1F  Factor Loadings from the Bifactor Analyses for Cisgender Participants, Assigned Male at Birth Participants, Assigned Female at Birth Participants, and Excluding Asexual, Not Sure, and Participants With a Different Sexual Orientation | | | | | | | | | | | | |
| --- | --- | --- | --- | --- | --- | --- | --- | --- | --- | --- | --- | --- |
|  | Cisgender | | | Assigned male at birth | | | Assigned female at birth | | | Excluding AND participants | | |
|  | General factor | Group factor 1 | Group factor 2 | General factor | Group factor 1 | Group factor 2 | General factor | Group factor 1 | Group factor 2 | General factor | Group factor 1 | Group factor 2 |
| 1. Imagine that you are walking through the hallway at school and a group of students is walking in your direction. When you pass them some of the students start to laugh. | .37 | .69 |  | .50 | .49 |  | .32 | .63 |  | .32 | .64 |  |
| 2. Imagine that you are instructed to work on a class assignment with a partner and no one wants to work with you. | .31 | .61 |  | .31 | .54 |  | .41 | .63 |  | .27 | .59 |  |
| 3. Imagine that you are walking on the streets with some friends. You get the feeling that some youth are following you. | .42 | .62 |  | .43 | .56 |  | .39 | .62 |  | .34 | .59 |  |
| 4. Imagine that a group of classmates are whispering together. They look into your direction and then continue to talk. | .38 | .52 |  | .37 | .45 |  | .39 | .59 |  | .38 | .53 |  |
| 5. Imagine that some of your classmates are celebrating a birthday of another classmate. You are not invited. | .37 | .48 |  | .42 | .39 |  | .43 | .38 |  | .32 | .41 |  |
| 6. Imagine that only you and a group of macho men are on a train. They look in your direction and laugh. | .25 | .72 |  | .29 | .63 |  | .32 | .62 |  | .23 | .65 |  |
| 7. Imagine that you are giving a presentation in class and a classmate laughs at you. | .43 | .40 |  | .42 | .26 |  | .45 | .42 |  | .39 | .39 |  |
| 8. Imagine that you are at work and a customer indicates they do not want to be helped by you. | .73 | -.16 |  | .67 | -.16 |  | .81 | -.15 |  | .78 | -.09 |  |
| 9. Imagine that you are watching a series with a LGBT character in it. One of your parents enters the room and says that there are too many gay people on TV. | .67 |  | .19 | .54 |  | .26 | .74 |  | .06 | .71 |  | -.05 |
| 10. Imagine that someone in your family makes a joke about LGBT people. | .69 |  | .46 | .56 |  | .52 | .81 |  | -.27 | .77 |  | -.30 |
| 11. Imagine that you are watching TV with your parents. There is a program/show about LGBT rights on TV. They change the channel. | .67 |  | -.09 | .69 |  | -.08 | .50 |  | .19 | .57 |  | .19 |
| 12. Imagine that you are at a family celebration with your same-sex partner. You notice your relatives looking at you, but they don't come over to talk to you. | .60 |  | -.32 | .61 |  | -.17 | .57 |  | .38 | .56 |  | .37 |
| 13. Imagine that you are on a date with someone of the same-sex at a restaurant. Your waiter provides you and your date with poor service. | .53 |  | -.46 | .48 |  | -.27 | .46 |  | .74 | .42 |  | .58 |
| 14. Imagine that you are walking into a shop holding hands with your same-sex partner. Other customers stare at you. | .33 |  | -.22 | .51 |  | -.34 | .40 |  | -.04 | .35 |  | .10 |
| AND = asexual, not sure, different sexual orientation. | | | | | | | | | | | | |

| Table S1G  *Descriptive Statistics and Correlations among Key Variables for Cisgender and Excluding Asexual, Not Sure, and Participants with a Different Sexual Orientation* | | | | | | | | | | | | | | | |  |
| --- | --- | --- | --- | --- | --- | --- | --- | --- | --- | --- | --- | --- | --- | --- | --- | --- |
|  | Cisgender | | | Excluding AND participants | | |  |  |  |  |  |  |  |  |  | |
|  | *M* | *SD* | Min-Max | *M* | *SD* | Min-max | 1. | 2. | 3. | 4. | 5. | 6. | 7. | 8. | 9. | |
| 1. SMA-RSS | 8.88 | 5.26 | 1.00 – 28.14 | 9.29 | 5.08 | 1.00 – 28.14 | - | **.28** | **.41** | **.27** | **.38** | **.31** | -.09 | **.33** | **.43** | |
| 2. Depressive symptoms | 2.13 | 0.58 | 1.00 – 3.70 | 2.21 | 0.61 | 1.00 – 3.90 | **.25** | - | **.64** | **.79** | **.42** | **.26** | -.08 | **.25** | **.41** | |
| 3. Anxiety symptoms | 2.17 | 0.63 | 1.00 – 4.00 | 2.20 | 0.60 | 1.00 – 4.00 | **.37** | **.64** | - | **.61** | **.35** | **.20** | -.02 | **.23** | **.46** | |
| 4. Psychosomatic complaints | 2.49 | 0.89 | 1.00 – 5.00 | 2.58 | 0.91 | 1.00 – 5.00 | **.24** | **.80** | **.61** | - | **.42** | **.22** | -.02 | **.23** | **.39** | |
| 5. Prejudice events | 1.69 | 0.60 | 1.00 – 4.60 | 1.80 | 0.67 | 1.00 – 4.60 | **.36** | **.43** | **.34** | **.42** | - | .07 | **.14** | **.13** | **.30** | |
| 6. Concealment | 2.92 | 1.08 | 1.00 – 5.00 | 2.86 | 1.08 | 1.00 – 5.00 | **.31** | **.29** | **.19** | **.28** | **.14** | - | **-.58** | **.44** | **.24** | |
| 7. Disclosure | 50.05 | 29.81 | 0.00 – 100.00 | 52.62 | 29.03 | 0.00 – 100.00 | -.07 | -.11 | -.06 | -.11 | .12 | **-.56** | - | **-.27** | -.07 | |
| 8. Internalized stigma | 2.06 | 0.79 | 1.00 – 4.60 | 1.99 | 0.75 | 1.00 – 4.6. | **.36** | **.28** | **.25** | **.23** | **.16** | **.44** | **-.30** | - | .**19** | |
| 9. General rejection sensitivity | 6.75 | 3.79 | 1.00 – 19.25 | 6.84 | 3.98 | 1.00 – 25.75 | **.33** | **.37** | **.39** | **.42** | **.26** | **.21** | -.09 | **.18** | - | |
| *Note.* Correlations under the diagonal are for cisgender participants and correlations above the diagonal are for gay, lesbian, bisexual, queer, and pansexual participants.  *M* = mean. *SD* = standard deviation. SMA-RSS = Sexual Minority Adolescent Rejection Sensitivity Scale. AND = asexual, not sure, different sexual orientation.  Bold numbers indicate p < .05 | | | | | | | | | | | | | | | |  |

| Table S1H  *Descriptive Statistics and Correlations among Key Variables for Assigned Male at Birth and Assigned Female at Birth Participants* | | | | | | | | | | | | | | | | |
| --- | --- | --- | --- | --- | --- | --- | --- | --- | --- | --- | --- | --- | --- | --- | --- | --- |
|  | Assigned male at birth | | | Assigned female at birth | | |  |  |  |  |  |  |  |  | |  |
|  | *M* | *SD* | Min-Max | *M* | *SD* | Min-max | 1. | 2. | 3. | 4. | 5. | 6. | 7. | | 8. | 9. |
| 1. SMA-RSS | 9.06 | 5.01 | 1.00 – 27.43 | 9.58 | 5.35 | 1.00 – 27.43 | - | **.36** | **.43** | **.40** | **.51** | **.31** | **-.12** | | **.39** | **.38** |
| 2. Depressive symptoms | 2.04 | 0.56 | 1.00 – 4.00 | 2.37 | 0.62 | 1.00 – 4.00 | .10 | - | **.65** | **.81** | **.44** | **.21** | .03 | | **.29** | **.41** |
| 3. Anxiety symptoms | 2.09 | 0.58 | 1.00 – 4.00 | 2.33 | 0.64 | 1.00 – 4.00 | **.24** | **.66** | - | **.62** | **.36** | **.17** | .04 | | **.23** | **.39** |
| 4. Psychosomatic complaints | 2.32 | 0.79 | 1.13 – 4.75 | 2.82 | 0.94 | 1.13 – 4.75 | .03 | **.71** | **.58** | - | **.45** | **.22** | .02 | | **.24** | **.39** |
| 5. Prejudice events | 1.86 | 0.67 | 1.00 – 4.60 | 1.80 | 0.67 | 1.00 – 4.60 | **.26** | **.41** | **.38** | **.50** | - | .02 | **.23** | | .11 | **.26** |
| 6. Concealment | 2.87 | 1.11 | 1.00 – 5.00 | 2.89 | 1.05 | 1.00 – 5.00 | **.26** | **.29** | .14 | **.22** | **.17** | - | **-.60** | | **.41** | **.26** |
| 7. Disclosure | 54.58 | 32.14 | 0.00 – 100.00 | 49.61 | 27.47 | 0.00 – 100.00 | .02 | -.17 | -.05 | .01 | .04 | **-.58** | - | | **-.27** | **-.13** |
| 8. Internalized stigma | 2.08 | 0.84 | 1.00 – 4.40 | 2.01 | 0.74 | 1.00 – 4.40 | **.21** | **.21** | **.21** | **.21** | .14 | **.40** | **-.32** | | - | **.19** |
| 9. General rejection sensitivity | 6.73 | 4.01 | 1.00 – 22.00 | 6.98 | 3.94 | 1.00 – 22.00 | **.40** | **.29** | **.39** | **.34** | **.30** | **.21** | -.04 | | .16 | - |
| *Note.* Correlations under the diagonal are for assigned male at birth participants and correlations above the diagonal are for assigned female participants.  *M* = mean. *SD* = standard deviation. SMA-RSS = Sexual Minority Adolescent Rejection Sensitivity Scale.  Bold numbers indicate p < .05 | | | | | | | | | | | | | | | | |

| Table S1I  *Descriptive Statistics and Correlations with Separate Factors of the SMA-RSS* | | | | | | | | | | | | | | |
| --- | --- | --- | --- | --- | --- | --- | --- | --- | --- | --- | --- | --- | --- | --- |
|  | *M* | *SD* | Min-Max | 1. | 2. | 3. | 4. | 5. | 6. | 7. | 8. | 9. | 10. |  |
| 1. SMA-RSS F1 | 6.90 | 5.12 | 1.00 – 32.12 | - |  |  |  |  |  |  |  |  |  |  |
| 2. SMA-RSS F2 | 12.72 | 7.23 | 1.00 – 36.00 | **.45** | - |  |  |  |  |  |  |  |  |  |
| 3. Depressive symptoms | 2.26 | 0.62 | 1.00 – 4.00 | **.20** | **.25** | - |  |  |  |  |  |  |  |  |
| 4. Anxiety symptoms | 2.25 | 0.63 | 1.00 – 4.00 | **.29** | **.32** | **.66** | - |  |  |  |  |  |  |  |
| 5. Psychosomatic complaints | 2.65 | 0.92 | 1.00 – 5.00 | **.22** | **.26** | **.79** | **.62** | - |  |  |  |  |  |  |
| 6. Prejudice events | 1.82 | 0.67 | 1.00 – 4.60 | **.44** | **.28** | **.40** | **.35** | **.43** | - |  |  |  |  |  |
| 7. Concealment | 2.88 | 1.07 | 1.00 – 5.00 | **.17** | **.32** | .22 | **.15** | **.21** | .07 | - |  |  |  |  |
| 8. Disclosure | 51.28 | 29.18 | 0.00 – 100.00 | .05 | **-.17** | -.05 | .00 | .00 | **.16** | **-.59** | - |  |  |  |
| 9. Internalized stigma | 2.04 | 0.77 | 1.00 – 4.60 | **.27** | **.28** | **.23** | **.20** | **.20** | **.12** | **.41** | **-.29** | - |  |  |
| 10. General rejection sensitivity | 6.89 | 3.96 | 1.00 – 25.75 | **.23** | **.39** | **.36** | **.38** | **.36** | **.27** | **.23** | -.10 | **.18** | - |  |
| *Note.* Correlations under the diagonal are for cisgender participants and correlations above the diagonal are for gay, lesbian, bisexual, queer, and pansexual participants.  *M* = mean. *SD* = standard deviation. SMA-RSS = Sexual Minority Adolescent Rejection Sensitivity Scale. AND = asexual, not sure, different sexual orientation.  Bold numbers indicate p < .05 | | | | | | | | | | | | | | |

| Table S1J  *Regression Analyses with the SMA-RSS Predicting Depressive Symptoms, Anxiety Symptoms, and Psychosomatic Complaints Over and Beyond Minority Stressors and General Rejection Sensitivity for Cisgender Participants, Assigned Male at Birth Participants, Assigned Female at Birth participants, and Excluding Asexual, Not Sure, and Participants with a Different Sexual Identity* | | | | | | | | | | | | | |
| --- | --- | --- | --- | --- | --- | --- | --- | --- | --- | --- | --- | --- | --- |
|  | Cisgender | | | Assigned male at birth | | | Assigned female at birth | | | Excluding AND participants | | | |
|  | Depressive symptoms | Anxiety symptoms | Psychosomatic complaints | Depressive symptoms | Anxiety symptoms | Psychosomatic complaints | Depressive symptoms | Anxiety symptoms | Psychosomatic complaints | Depressive symptoms | Anxiety symptoms | Psychosomatic complaints | |
|  | ß (SE) | ß (SE) | ß (SE) | ß (SE) | ß (SE) | ß (SE) | ß (SE) | ß (SE) | ß (SE) | ß (SE) | ß (SE) | ß (SE) | |
| Prejudice events | **0.39** (0.06) | **0.24** (0.06) | **0.39** (0.06) | **0.41** (0.08) | **0.35** (0.09) | **0.51** (0.07) | **0.35** (0.07) | **0.19** (0.06) | **0.36** (0.07) | **0.37** (0.05) | **0.24** (0.06) | **0.38** (0.05) | |
| SMA-RSS | 0.11 (0.07) | **0.29** (0.07) | 0.11 (0.07) | -0.01 (0.09) | 0.15 (0.09) | -0.12 (0.09) | **0.18** (0.07) | **0.35** (0.07) | **0.23** (0.06) | **0.15** (0.06) | **0.33** (0.05) | **0.14** (0.06) | |
|  |  |  |  |  |  |  |  |  |  |  |  |  | |
| Concealment | **0.24** (0.07) | 0.09 (0.07) | **0.22** (0.06) | **0.31** (0.09) | 0.10 (0.09) | **0.21** (0.09) | 0.11 (0.06) | 0.04 (0.06) | 0.08 (0.07) | **0.19** (0.06) | 0.08 (0.06) | **0.14** (0.06) | |
| SMA-RSS | **0.18** (0.08) | **0.34** (0.08) | **0.18** (0.07) | 0.02 (0.10) | **0.22** (0.11) | -0.05 (0.11) | **0.32** (0.06) | **0.42** (0.06) | **0.37** (0.06) | **0.23** (0.06) | **0.39** (0.05) | **0.24** (0.06) | |
|  |  |  |  |  |  |  |  |  |  |  |  |  |  |
| Disclosure | -0.10 (0.06) | -0.04 (0.06) | -0.09 (0.06) | -0.18 (0.09) | -0.05 (0.09) | 0.02 (0.09) | 0.07 (0.05) | 0.10 (0.05) | 0.09 (0.06) | -0.06 (0.05) | 0.01 (0.05) | 0.01 (0.06) | |
| SMA-RSS | **0.24** (0.07) | **0.37** (0.07) | **0.24 (0.06)** | 0.10 (0.10) | **0.24** (0.10) | 0.01 (011) | **0.36** (0.05) | **0.44** (0.06) | **0.41** (0.05) | **0.28** (0.05) | **0.41** (0.05) | **0.28** (0.06) | |
|  |  |  |  |  |  |  |  |  |  |  |  |  |  |
| Internalized stigma | **0.22** (0.07) | 0.10 (0.06) | **0.15** (0.07) | 0.20 (0.09) | 0.16 (0.08) | **0.20** (0.08) | **0.18** (0.06) | 0.07 (0.06) | 0.08 (0.06) | **0.17** (0.06) | 0.11 (0.05) | **0.15** (0.06) | |
| SMA-RSS | **0.17** (0.07) | **0.34** (0.06) | **0.19** (0.08) | 0.06 (0.10) | **0.21** (0.11) | -0.03 (0.11) | **0.29** (0.06) | **0.41** (0.06) | **0.37** (0.06) | **0.23** (0.06) | **0.38** (0.05) | **0.23** (0.06) | |
|  |  |  |  |  |  |  |  |  |  |  |  |  |  |
| General rejection sensitivity | **0.33** (0.07) | **0.31** (0.07) | **0.39** (0.06) | **0.28** (0.08) | **0.35** (0.09) | **0.38** (0.09) | **0.33** (0.06) | **0.27** (0.06) | **0.28** (0.06) | **0.35** (0.05) | **0.36** (0.05) | **0.33** (0.05) | |
| SMA-RSS | **0.15** (0.08) | **0.28** (0.08) | 0.12 (0.07) | 0.00 (0.10) | 0.12 (0.10) | -0.15 (0.11) | **0.24** (0.07) | **0.34** (0.07) | **0.30** (0.06) | **0.15** (0.06) | **0.28** (0.06) | **0.14** **(0.07)** | |
|  |  |  |  |  |  |  |  |  |  |  |  |  | |
| Prejudice events | **0.36** (0.06) | **0.21** (0.06) | **0.35** (0.06) | **0.36** (0.09) | **0.29** (0.10) | **0.42** (0.08) | **0.33** (0.07) | **0.15** (0.07) | **0.33** (0.07) | **0.34** (0.05) | **0.19** (0.06) | **0.34** (0.06) | |
| Concealment | 0.13 (0.07) | 0.00 (0.08) | 0.14 (0.07) | 0.23 (0.13) | -0.04 (0.14) | **0.21** (0.09) | **0.15** (0.07) | 0.09 (0.07) | 0.15 (0.08) | 0.13 (0.06) | 0.04 (0.07) | 0.13 (0.07) | |
| Disclosure | -0.03 (0.06) | -0.03 (0.07) | -0.03 (0.07) | -0.01 (0.13) | -0.02 (0.13) | **0.19** (0.09) | 0.13 (0.06) | **0.16** (0.07) | 0.13 (0.07) | -0.02 (0.05) | 0.04 (0.06) | 0.06 (0.06) | |
| Internalized stigma | **0.14** (0.08) | 0.11 (0.07) | 0.07 (0.06) | 0.07 (0.09) | 0.13 (0.09) | 0.14 (0.07) | **0.19** (0.07) | 0.09 (0.07) | 0.09 (0.07) | **0.11** (0.06) | 0.09 (0.06) | **0.11** (0.06) | |
| General rejection sensitivity | **0.25** (0.07) | **0.28** (0.07) | **0.32** (0.06) | 0.17 (0.08) | **0.28** (0.09) | **0.25** (0.08) | **0.29** (0.06) | **0.27** (0.06) | **0.25** (0.06) | **0.28** (0.06) | **0.33** (0.05) | **0.26** (0.06) | |
| SMA-RSS | -0.05 (0.08) | **0.19** (0.08) | -0.05 (0.07) | -0.13 (0.09) | 0.06 (0.10) | **-0.27** (0.08) | -0.01 (0.07) | **0.24** (0.08) | 0.09 (0.07) | -0.03 (0.06) | **0.19** (0.06) | -0.02 (0.07) | |
| SMA-RSS = Sexual Minority Adolescent Rejection Sensitivity Scale. AND = asexual, not sure, different sexual identity.  Bold numbers indicate *p* < .05 | | | | | | | | | | | | | |

| Table S1K  *Regression Analyses with Separate Factors of the SMA-RSS Predicting Depressive Symptoms, Anxiety Symptoms, and Psychosomatic Complaints Over and Beyond Minority Stressors and General Rejection Sensitivity* | | | | | | | |
| --- | --- | --- | --- | --- | --- | --- | --- |
|  | Cisgender | | | | | | |
|  | Depressive symptoms | | | Anxiety symptoms | | Psychosomatic complaints | |
|  | ß | SE | | ß | SE | ß | SE |
| Prejudice events | **0.37** | 0.05 | | **0.26** | 0.05 | **0.41** | 0.05 |
| SMA-RSS F1 | -0.05 | 0.06 | | 0.09 | 0.06 | -0.04 | 0.06 |
| SMA-RSS F2 | **0.17** | 0.05 | | **0.22** | 0.05 | **0.17** | 0.06 |
|  |  |  | |  |  |  |  |
| Concealment | **0.17** | 0.05 | | 0.06 | 0.05 | 0.12 | 0.06 |
| SMA-RSS F1 | 0.10 | 0.06 | | **0.19** | 0.06 | 0.13 | 0.06 |
| SMA-RSS F2 | 0.16 | 0.06 | | **0.22** | 0.06 | **0.17** | 0.06 |
|  |  |  | |  |  |  |  |
| Disclosure | -0.03 | 0.05 | | 0.03 | 0.05 | 0.04 | 0.05 |
| SMA-RSS F1 | 0.10 | 0.06 | | **0.18** | 0.06 | 0.12 | 0.06 |
| SMA-RSS F2 | **0.20** | 0.06 | | **0.25** | 0.05 | **0.21** | 0.06 |
|  |  |  | |  |  |  |  |
| Internalized stigma | **0.17** | 0.05 | | 0.10 | 0.05 | 0.12 | 0.05 |
| SMA-RSS F1 | 0.07 | 0.06 | | **0.17** | 0.06 | 0.11 | 0.07 |
| SMA-RSS F2 | **0.18** | 0.06 | | **0.22** | 0.05 | **0.18** | 0.06 |
|  |  |  | |  |  |  |  |
| General rejection sensitivity | **0.31** | 0.05 | | **0.30** | 0.05 | **0.30** | 0.05 |
| SMA-RSS F1 | 0.08 | 0.06 | | **0.18** | 0.06 | 0.11 | 0.07 |
| SMA-RSS F2 | 0.10 | 0.06 | | 0.14 | 0.06 | 0.10 | 0.07 |
|  |  |  | |  |  |  |  |
| Prejudice events | **0.34** | 0.05 | | **0.21** | 0.06 | **0.37** | 0.05 |
| Concealment | 0.14 | 0.06 | | 0.04 | 0.07 | 0.14 | 0.06 |
| Disclosure | 0.04 | 0.06 | | 0.06 | 0.06 | 0.10 | 0.06 |
| Internalized stigma | 0.13 | 0.06 | | 0.10 | 0.06 | 0.10 | 0.06 |
| General rejection sensitivity | **0.23** | 0.05 | | **0.27** | 0.05 | **0.23** | 0.05 |
| SMA-RSS F1 | -0.08 | 0.06 | | 0.07 | 0.07 | -0.07 | 0.06 |
| SMA-RSS F2 | 0.04 | | 0.06 | 0.12 | 0.06 | 0.06 | 0.06 |
| SMA-RSS = Sexual Minority Adolescent Rejection Sensitivity Scale.  Bold numbers indicate *p* < .05 | | | | | | | |

**The Sexual Minority Adolescents Rejection sensitivity Scale (SMA-RSS) Dutch**

| **Scoring procedure** |
| --- |
| Om een score voor de SMA-RSS te krijgen voor een participant moet je:   1. Voor elke situatie de score op vraag 1 en vraag 2 met elkaar vermenigvuldigen. 2. De product scores van alle 14 vragen bij elkaar op te tellen en vervolgens delen door 14. |

| **Introductie tekst** |
| --- |
| We gaan nu een aantal situaties aan je voorleggen. Deze beginnen allemaal met "Stel je voor..." Probeer zo goed mogelijk in te denken hoe de situatie voor jou zou zijn. Bij elke situatie zijn er steeds twee vragen die je moet beantwoorden. Sommige situaties gaan over een partner van hetzelfde geslacht, je kunt dit ook lezen als een partner van hetzelfde gender. |

| **Situatie 1. Stel je voor, je loopt door de gang op school en een groepje leerlingen loopt jouw kant op. Als je langsloopt hoor je een aantal van hen lachen.** | | | | | | | | | |
| --- | --- | --- | --- | --- | --- | --- | --- | --- | --- |
| Vraag 1. Hoe bezorgd of bang zou je zijn dat ze lachen vanwege jouw seksuele oriëntatie? | Heel erg onbezorgd | | | | | Heel er bezorgd | | | |
|  | 1 | 2 | | 3 | | 4 | | 5 | 6 |
| Vraag 2. Hoe waarschijnlijk is het dat ze lachen vanwege jouw seksuele oriëntatie? | Heel erg onwaarschijnlijk | | | | | Heel erg waarschijnlijk | | | |
|  | 1 | | 2 | | 3 | 4 | 5 | | 6 |

| **Situatie 2. Stel je voor, voor een opdracht op school moet je samenwerken, maar niemand wil met jou samenwerken.** | | | | | | | | | |
| --- | --- | --- | --- | --- | --- | --- | --- | --- | --- |
| Vraag 1. Hoe bezorgd of bang zou je zijn dat niemand met jou wil samenwerken vanwege jouw seksuele oriëntatie? | Heel erg onbezorgd | | | | | Heel er bezorgd | | | |
|  | 1 | 2 | | 3 | | 4 | | 5 | 6 |
| Vraag 2. Hoe waarschijnlijk is het dat niemand met jou wil samenwerken vanwege jouw seksuele oriëntatie? | Heel erg onwaarschijnlijk | | | | | Heel erg waarschijnlijk | | | |
|  | 1 | | 2 | | 3 | 4 | 5 | | 6 |

| **Situatie 3. Stel je voor, je loopt op straat met vrienden. Jullie hebben het gevoel dat jullie worden gevolgd door een groepje jongeren.** | | | | | | | | | |
| --- | --- | --- | --- | --- | --- | --- | --- | --- | --- |
| Vraag 1. Hoe bezorgd of bang zou je zijn dat jullie worden gevolgd vanwege jouw seksuele oriëntatie? | Heel erg onbezorgd | | | | | Heel er bezorgd | | | |
|  | 1 | 2 | | 3 | | 4 | | 5 | 6 |
| Vraag 2. Hoe waarschijnlijk is het dat jullie worden gevolgd vanwege jouw seksuele oriëntatie? | Heel erg onwaarschijnlijk | | | | | Heel erg waarschijnlijk | | | |
|  | 1 | | 2 | | 3 | 4 | 5 | | 6 |

| **Situatie 4. Stel je voor, een groepje klasgenoten is aan het fluisteren met elkaar. Ze kijken even jouw kant op en praten daarna verder.** | | | | | | | | | |
| --- | --- | --- | --- | --- | --- | --- | --- | --- | --- |
| Vraag 1. Hoe bezorgd of bang zou je zijn dat ze over jou fluisteren vanwege jouw seksuele oriëntatie? | Heel erg onbezorgd | | | | | Heel er bezorgd | | | |
|  | 1 | 2 | | 3 | | 4 | | 5 | 6 |
| Vraag 2. Hoe waarschijnlijk is het dat ze over jou fluisteren vanwege jouw seksuele oriëntatie? | Heel erg onwaarschijnlijk | | | | | Heel erg waarschijnlijk | | | |
|  | 1 | | 2 | | 3 | 4 | 5 | | 6 |

| **Situatie 5. Stel je voor, je klasgenoten vieren een verjaardag, maar jij bent niet uitgenodigd.** | | | | | | | | | |
| --- | --- | --- | --- | --- | --- | --- | --- | --- | --- |
| Vraag 1. Hoe bezorgd of bang zou je zijn dat je niet werd uitgenodigd vanwege jouw seksuele oriëntatie? | Heel erg onbezorgd | | | | | Heel er bezorgd | | | |
|  | 1 | 2 | | 3 | | 4 | | 5 | 6 |
| Vraag 2. Hoe waarschijnlijk is het dat je niet werd uitgenodigd vanwege jouw seksuele oriëntatie? | Heel erg onwaarschijnlijk | | | | | Heel erg waarschijnlijk | | | |
|  | 1 | | 2 | | 3 | 4 | 5 | | 6 |

| **Situatie 6. Stel je voor, je zit in een trein en bij jou in de coupé zit een groepje stoere mannen. Ze kijken in jouw richting en beginnen te lachen.** | | | | | | | | | |
| --- | --- | --- | --- | --- | --- | --- | --- | --- | --- |
| Vraag 1. Hoe bezorgd of bang zou je zijn dat ze lachen vanwege jouw seksuele oriëntatie? | Heel erg onbezorgd | | | | | Heel er bezorgd | | | |
|  | 1 | 2 | | 3 | | 4 | | 5 | 6 |
| Vraag 2. Hoe waarschijnlijk is het dat ze lachen vanwege jouw seksuele oriëntatie? | Heel erg onwaarschijnlijk | | | | | Heel erg waarschijnlijk | | | |
|  | 1 | | 2 | | 3 | 4 | 5 | | 6 |

| **Situatie 7. Stel je voor, je bent aan het presenteren tijdens een les en een klasgenoot lacht je uit.** | | | | | | | | | |
| --- | --- | --- | --- | --- | --- | --- | --- | --- | --- |
| Vraag 1. Hoe bezorgd of bang zou je zijn dat je klasgenoot je uitlacht vanwege jouw seksuele oriëntatie? | Heel erg onbezorgd | | | | | Heel er bezorgd | | | |
|  | 1 | 2 | | 3 | | 4 | | 5 | 6 |
| Vraag 2. Hoe waarschijnlijk is het dat je klasgenoot je uitlacht vanwege jouw seksuele oriëntatie? | Heel erg onwaarschijnlijk | | | | | Heel erg waarschijnlijk | | | |
|  | 1 | | 2 | | 3 | 4 | 5 | | 6 |

| **Situatie 8. Stel je voor, op je werk zegt een klant niet door jou te willen worden geholpen.** | | | | | | | | | |
| --- | --- | --- | --- | --- | --- | --- | --- | --- | --- |
| Vraag 1. Hoe bezorgd of bang zou je zijn dat de klant niet door jou wil worden geholpen vanwege jouw seksuele oriëntatie? | Heel erg onbezorgd | | | | | Heel er bezorgd | | | |
|  | 1 | 2 | | 3 | | 4 | | 5 | 6 |
| Vraag 2. Hoe waarschijnlijk is het dat de klant niet door jou wil worden geholpen vanwege jouw seksuele oriëntatie? | Heel erg onwaarschijnlijk | | | | | Heel erg waarschijnlijk | | | |
|  | 1 | | 2 | | 3 | 4 | 5 | | 6 |

| **Situatie 9. Stel je voor, je kijkt een serie waar een LHBT-personage in zit. Een van je ouders komt de kamer in lopen en zegt dat er te veel homo’s op TV zijn.** | | | | | | | | | |
| --- | --- | --- | --- | --- | --- | --- | --- | --- | --- |
| Vraag 1. Hoe bezorgd of bang zou je zijn dat je minder wordt geaccepteerd vanwege jouw seksuele oriëntatie? | Heel erg onbezorgd | | | | | Heel er bezorgd | | | |
|  | 1 | 2 | | 3 | | 4 | | 5 | 6 |
| Vraag 2. Hoe waarschijnlijk is het dat je minder wordt geaccepteerd vanwege jouw seksuele oriëntatie? | Heel erg onwaarschijnlijk | | | | | Heel erg waarschijnlijk | | | |
|  | 1 | | 2 | | 3 | 4 | 5 | | 6 |

| **Situatie 10. Stel je voor, iemand in je familie maakt een grap over LHBT’ers.** | | | | | | | | | |
| --- | --- | --- | --- | --- | --- | --- | --- | --- | --- |
| Vraag 1. Hoe bezorgd of bang zou je zijn dat deze persoon jou niet accepteert vanwege jouw seksuele oriëntatie? | Heel erg onbezorgd | | | | | Heel er bezorgd | | | |
|  | 1 | 2 | | 3 | | 4 | | 5 | 6 |
| Vraag 2. Hoe waarschijnlijk is het dat deze persoon jou niet accepteert vanwege jouw seksuele oriëntatie? | Heel erg onwaarschijnlijk | | | | | Heel erg waarschijnlijk | | | |
|  | 1 | | 2 | | 3 | 4 | 5 | | 6 |

| **Situatie 11. Stel je voor, je kijkt samen met je ouders TV. Op TV is een item over LHBT-rechten. Ze veranderen van zender.** | | | | | | | | | |
| --- | --- | --- | --- | --- | --- | --- | --- | --- | --- |
| Vraag 1. Hoe bezorgd of bang zou je zijn dat ze je minder accepteren vanwege jouw seksuele oriëntatie? | Heel erg onbezorgd | | | | | Heel er bezorgd | | | |
|  | 1 | 2 | | 3 | | 4 | | 5 | 6 |
| Vraag 2. Hoe waarschijnlijk is het dat ze je minder accepteren vanwege jouw seksuele oriëntatie? | Heel erg onwaarschijnlijk | | | | | Heel erg waarschijnlijk | | | |
|  | 1 | | 2 | | 3 | 4 | 5 | | 6 |

| **Situatie 12. Stel je voor, met je partner van hetzelfde geslacht ben je op een familiefeest. Je ziet een aantal familieleden naar jullie kijken, maar ze komen niet met jullie praten.** | | | | | | | | | |
| --- | --- | --- | --- | --- | --- | --- | --- | --- | --- |
| Vraag 1. Hoe bezorgd of bang zou je zijn dat ze niet naar jou toe komen vanwege jouw seksuele oriëntatie? | Heel erg onbezorgd | | | | | Heel er bezorgd | | | |
|  | 1 | 2 | | 3 | | 4 | | 5 | 6 |
| Vraag 2. Hoe waarschijnlijk is het dat ze niet naar jou toe komen vanwege jouw seksuele oriëntatie? | Heel erg onwaarschijnlijk | | | | | Heel erg waarschijnlijk | | | |
|  | 1 | | 2 | | 3 | 4 | 5 | | 6 |

| **Situatie 13. Stel je voor, je bent op een date met iemand van hetzelfde geslacht. De ober doet onbeleefd naar jullie.** | | | | | | | | | |
| --- | --- | --- | --- | --- | --- | --- | --- | --- | --- |
| Vraag 1. Hoe bezorgd of bang zou je zijn dat er onbeleefd werd gedaan vanwege jouw seksuele oriëntatie? | Heel erg onbezorgd | | | | | Heel er bezorgd | | | |
|  | 1 | 2 | | 3 | | 4 | | 5 | 6 |
| Vraag 2. Hoe waarschijnlijk is het dat er onbeleefd werd gedaan vanwege jouw seksuele oriëntatie? | Heel erg onwaarschijnlijk | | | | | Heel erg waarschijnlijk | | | |
|  | 1 | | 2 | | 3 | 4 | 5 | | 6 |

| **Situatie 14. Stel je voor, je loopt hand in hand een winkel binnen met je partner van hetzelfde geslacht. Andere klanten staren naar jullie.** | | | | | | | | | |
| --- | --- | --- | --- | --- | --- | --- | --- | --- | --- |
| Vraag 1. Hoe bezorgd of bang zou je zijn dat ze naar jou staren vanwege jouw seksuele oriëntatie? | Heel erg onbezorgd | | | | | Heel er bezorgd | | | |
|  | 1 | 2 | | 3 | | 4 | | 5 | 6 |
| Vraag 2. Hoe waarschijnlijk is het dat ze naar jou staren vanwege jouw seksuele oriëntatie? | Heel erg onwaarschijnlijk | | | | | Heel erg waarschijnlijk | | | |
|  | 1 | | 2 | | 3 | 4 | 5 | | 6 |
